# *BioM2*: biologically informed multi-stage machine learning for phenotype prediction using omics data

**DOI:** 10.1093/bib/bbae384

**Published:** 2024-08-10

**Authors:** Shunjie Zhang, Pan Li, Shenghan Wang, Jijun Zhu, Zhongting Huang, Fuqiang Cai, Sebastian Freidel, Fei Ling, Emanuel Schwarz, Junfang Chen

**Affiliations:** School of Biology and Biological Engineering, South China University of Technology, Guangzhou, China; Center for Intelligent Medicine, Greater Bay Area Institute of Precision Medicine (Guangzhou), School of Life Sciences, Fudan University, No. 6, 2nd Nanjiang Road, Nansha District, 511462 Guangzhou, China; Center for Intelligent Medicine, Greater Bay Area Institute of Precision Medicine (Guangzhou), School of Life Sciences, Fudan University, No. 6, 2nd Nanjiang Road, Nansha District, 511462 Guangzhou, China; Center for Intelligent Medicine, Greater Bay Area Institute of Precision Medicine (Guangzhou), School of Life Sciences, Fudan University, No. 6, 2nd Nanjiang Road, Nansha District, 511462 Guangzhou, China; Center for Intelligent Medicine, Greater Bay Area Institute of Precision Medicine (Guangzhou), School of Life Sciences, Fudan University, No. 6, 2nd Nanjiang Road, Nansha District, 511462 Guangzhou, China; School of Biology and Biological Engineering, South China University of Technology, Guangzhou, China; Hector Institute for Artificial Intelligence in Psychiatry, Central Institute of Mental Health, Medical Faculty Mannheim, Heidelberg University, M7, Mannheim 68161, Germany; Department of Psychiatry and Psychotherapy, Central Institute of Mental Health, Medical Faculty Mannheim, Heidelberg University, J5, Mannheim 68159, Germany; School of Biology and Biological Engineering, South China University of Technology, Guangzhou, China; Hector Institute for Artificial Intelligence in Psychiatry, Central Institute of Mental Health, Medical Faculty Mannheim, Heidelberg University, M7, Mannheim 68161, Germany; Department of Psychiatry and Psychotherapy, Central Institute of Mental Health, Medical Faculty Mannheim, Heidelberg University, J5, Mannheim 68159, Germany; Center for Intelligent Medicine, Greater Bay Area Institute of Precision Medicine (Guangzhou), School of Life Sciences, Fudan University, No. 6, 2nd Nanjiang Road, Nansha District, 511462 Guangzhou, China; Center for Evolutionary Biology, School of Life Sciences, Fudan University, Shanghai, China

**Keywords:** BioM2, machine learning, phenotype prediction, DNA methylome, transcriptome, Gene Ontology

## Abstract

Navigating the complex landscape of high-dimensional omics data with machine learning models presents a significant challenge. The integration of biological domain knowledge into these models has shown promise in creating more meaningful stratifications of predictor variables, leading to algorithms that are both more accurate and generalizable. However, the wider availability of machine learning tools capable of incorporating such biological knowledge remains limited. Addressing this gap, we introduce *BioM2*, a novel R package designed for biologically informed multistage machine learning. *BioM2* uniquely leverages biological information to effectively stratify and aggregate high-dimensional biological data in the context of machine learning. Demonstrating its utility with genome-wide DNA methylation and transcriptome-wide gene expression data, *BioM2* has shown to enhance predictive performance, surpassing traditional machine learning models that operate without the integration of biological knowledge. A key feature of *BioM2* is its ability to rank predictor variables within biological categories, specifically Gene Ontology pathways. This functionality not only aids in the interpretability of the results but also enables a subsequent modular network analysis of these variables, shedding light on the intricate systems-level biology underpinning the predictive outcome. We have proposed a biologically informed multistage machine learning framework termed *BioM2* for phenotype prediction based on omics data. *BioM2* has been incorporated into the *BioM2* CRAN package (https://cran.r-project.org/web/packages/BioM2/index.html).

## Introduction

Recent advancements in high-throughput technologies have led to an unprecedented increase in the availability of large-scale omics data, providing new opportunities for exploring complex illness and traits [1–3]. Despite the technological strides, the challenge of extracting biologically meaningful and predictive signatures from high-dimensional biological data persists [4–6], even with the widespread application of machine learning techniques [7–10] and advanced methodologies like deep learning [11–14].

In terms of developing explainable machine learning models, three key terms emerge: transparency, interpretability and explainability [15]. Transparency primarily refers to the machine learning model itself, interpretability involves the model together with data, and explainability extends to encompass the model, the data and the important aspect of human involvement. Central to the notion of explainability is the concept of informed machine learning, which advocates for explicit integration of domain knowledge into the machine learning framework [16]. Besides the necessity for large sample sizes, it is becoming increasingly clear that modern machine learning techniques applied to complex biological problems would substantially profit from the inclusion of biological domain knowledge [17–24]. Genome-wide functional studies based on co-expression or gene regulatory networks [25], biological pathways or functionally related genes [26] have already provided valuable insights into processes associated with illnesses and complex traits. This knowledge base presents a significant opportunity to invert traditional analytical paradigms and develop biologically informed machine learning tools that can derive mechanistically important signatures from high-dimensional data. Such tools may show a higher degree of biological reproducibility and resilience against the noise inherent in these datasets. In the field of oncology, there has been a notable increase in the utilization of deep learning methods that integrate biological domain knowledge [17], for instance, the interpretable Convolutional Neural Network model (PathCNN) [18], which leverages pathway information from the Kyoto Encyclopedia of Genes and Genomes (KEGG) database for survival prediction and pathway analysis in glioblastoma, and a deep learning model utilizing thousands of curated biological pathways from the Reactome database to advance the discovery of insights related to prostate cancer [19]. Additionally, a wide array of studies have incorporated biological knowledge into machine learning applications across different omics data modalities [20–24]. One example is our previously described biologically informed multistage machine learning (BioMM) model, which is based on genome-wide DNA methylation data [23, 27].

Here, we developed a new R package named *BioM2* that can be readily used as a versatile tool for machine learning applications while integrating prior biological information. The key functionalities of BioM2 include the following: (a) phenotype prediction using whole-genome DNA methylation data and genome-wide gene expression data; (b) intrinsic ranking of outcome-associated functional patterns; (c) functional patterns that can be used for biological stratification on an individual subject basis; (d) modularization of functional patterns and subsequent network analysis of these modules; (e) various choice of conventional machine learning models that can be integrated within the *BioM2* framework.

## Materials and methods

### Genome-wide DNA methylation and gene expression data

In this work, we used two real-world example datasets (GSE198904 and GSE46743) downloaded from the GEO database [28], which have been preprocessed according to the procedure described in references [29, 30]. The first dataset (GSE198904) comprised genome-wide DNA methylation profiles from two cohorts of major depressive disorder (MDD) case–control studies. We focused on one cohort within this dataset, which included 429 whole blood samples. Specifically, it contained data from 361 MDD cases and 68 healthy controls. We matched 68 MDD cases and 68 healthy controls from a pool of 429 whole blood samples. This match was based on potential confounding factors including gender, age, blood cell composition, population structure and smoking score, using the propensity score matching method. We employed the “nearest” method with a one-to-one ratio through the matchit function from the MatchIt R package (version 4.5.4) to ensure a balanced comparison between groups [31]. This approach ensured a balanced comparison between cases with depressive disorders and healthy controls. Furthermore, we adjusted the genome-wide DNA methylation data for these confounding factors. The second dataset (GSE46743) consisted of genome-wide gene expression data from 160 male whole blood samples, encompassing 69 MDD cases and 91 healthy controls. Employing the same propensity score matching method as with the first dataset, we selected 69 baseline cases and an equal number of baseline controls for our analysis, matching based on age and body mass index.

### Gene and pathway assignment

For the mapping of genes corresponding to 5′-cytosine-phosphodiester bond-guanine-3′ (CpG) sites, we utilized annotations from the Illumina Infinium MethylationEPIC BeadChip. Similarly, the annotation of probes associated with gene expression data was derived from the Illumina HumanHT-12 V3.0 Expression BeadChip. Subsequently, we utilized the *GO.db* (version 3.16.0) and *org.Hs.eg.db* (version 3.16) R packages [32, 33] to retrieve annotated gene sets pertaining to biological processes from the Gene Ontology (GO) database [34], referred to as “pathways.” A total of 3932 GO pathways, each comprising between 20 and 200 genes with at least 10 CpGs per pathway for inclusion, were used for further analysis, whereas 3700 GO pathways, each with 20 and 200 genes, were kept for genome-wide gene expression data, analogous to the choice of pathway size used in genome-wide association study [26]. For both data modalities, the number of genes in each pathway was restricted for downstream analysis as too small pathways have insufficient predictive power due to a limited number of predictors, and too large pathways often lead to computationally demanding analyses and may encompass biologically nonspecific associations. Only autosomal CpGs or genes were used in this analysis to avoid complications arising from X-chromosome inactivation or the presence of an additional X chromosome in females. In total, 372 634 CpGs were mapped to gene ontological pathways in our DNA methylation dataset, with an additional 356 928 CpGs that were not mapped to any specific pathways. Among these, 221 707 CpGs were found to lack any mapping relationship and are strictly defined as unmapped features. A total of 9552 gene probes aligned with gene ontological pathways in our gene expression dataset, while 4616 were not mapped to pathways. Among these, 959 gene probes were identified as lacking any mapping relationship and are strictly defined as unmapped features.

### *BioM2* architecture

**Figure 1:**
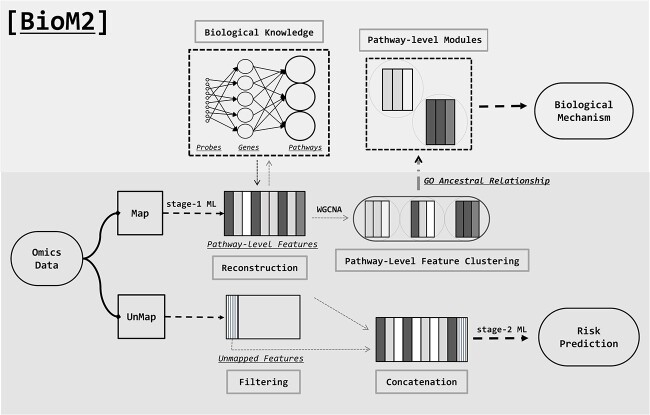
The architecture of *BioM2*. Omics data are used as input to create pathway features through supervised machine learning, including features with biological mapping relationships. Unmapped features are partially filtered and then concatenated for final prediction. Additionally, *BioM2* enables exploration of intermediate pathway-level features, which are clustered into modules using WGCNA. Significant modules are identified based on GO pathway relationships, enhancing our understanding of disease-related biological mechanisms.

A schematic illustration of the *BioM2*’s architecture, designed for binary classification in risk prediction and elucidating the biological mechanisms underlying diseases. This architecture is applicable to both genome-wide DNA methylation and gene expression data in Fig. 1. The input omics data (genome-wide DNA methylation data or gene expression data) are used as input, and features in the data are separated into two distinct types based on the provided annotation information. The first type consists of features that align with biological pathways, as identified through the GO database. These features are subjected to supervised machine learning techniques using resampling approach to generate pathway-level features. The second type includes features that do not map to any specific biological pathway and have not been the focus of biological interpretation, but may still contribute to disease prediction. These features undergo a feature selection process to distill relevant information. The pathway-level features and the refined non-pathway features are then amalgamated to form a second-stage dataset. This merged dataset is subsequently used as the input for the final predictive model in a supervised machine learning framework. Furthermore, *BioM2* facilitates an in-depth exploration of intermediate products, specifically the pathway-level features. These features are clustered into “pathway modules” using a Weighted Gene Co-expression Network Analysis (WGCNA) approach [35]. To gain insights into the biological mechanisms and disease-associated difference, we screen for biologically significant modules based on their ancestral relationships within the GO pathways. This approach allows for a nuanced understanding of the underlying biological processes and their potential implications in disease manifestation and progression.

### *BioM2* algorithm

The *BioM2* framework is structured in a multilayered architecture, encompassing an input layer, two intermediary layers (stage 1 and stage 2) and a final output layer. The input layer is the genome-wide DNA methylation data, which is then passed to the stage-1 layer (i.e. stage-1 data) by incorporating biological metadata information. At this stage, biological metadata is incorporated into the input data, transforming it into stage-1 data. This process involves mapping the CpG sites to specific biological pathways based on gene annotations, effectively categorizing the methylation data into distinct pathways. The data from the stage-1 layer undergo a further transformation. Utilizing machine learning techniques, pathway-level features are generated from the methylation data associated with each pathway. Concurrently, “unmapped features,” which are not aligned with any pathway, are identified and refined through feature selection. These pathway-level and unmapped features together constitute the stage-2 data, which encapsulates the interdependencies among various pathways. The final layer of the algorithm is the output layer, where the prediction results are generated. This is achieved by applying a supervised machine learning model to the stage-2 data.

From a machine learning perspective, the BioM2 algorithm operates in two major stages. The first stage involves compressing the variables of the original dataset (stage-1 data) into a much lower-dimensional form (stage-2 data), which includes pathway-level features. This compression is achieved using supervised learning models, where predictions derived from machine learning during resampling are stored, or through algorithms applied to an independent test set. In the second stage, the stage-2 data are used to build a supervised model, culminating in predictions at the output layer. This process effectively captures and utilizes the intricate relationships between different biological pathways. A comprehensive and detailed algorithmic procedure is described in Algorithm 1.

**Algorithm 1:** *BioM2* algorithm.

**Input:** 

1. K: total number of annotated pathways.
2. D_k_: stage-1 training dataset D_k_ = {(${\mathrm{x}}_1^k$,…, ${\mathrm{x}}_M^k$)^T^, Y_D_} with M samples for every k = 1,…,K, i.e. ${\mathrm{x}}_i^k$∈R(n_k_*1) and a label vector Y_D_∈R(M*1).
3. p: total number of features unmapped to the annotated pathway.
4.
${X}_{pM}$ : *p* unmapped features screened by feature selection method with M samples, i.e. ${X}_{pM}$∈R(M*p).
5. f^1^: stage-1 machine learning model.
6. f^2^: stage-2 machine learning model.

**Output:** 

1.
${\hat{\mathrm{Y}}}_{kM}^{(1)}$ : stage-1 cross-validation prediction score with M samples for k^th^ pathway, i.e. ${\hat{\mathrm{Y}}}_{kM}^{(1)}$∈R(M*1).
2. D^2^: stage-2 training data D^2^ = (${\hat{\mathrm{Y}}}_{1M}^{(1)}$,…, ${\hat{\mathrm{Y}}}_{KM}^{(1)}$, ${X}_{pM}$, Y_D_), with M samples and a label vector Y_D_.
3. Err_cv_: error metric for the cross-validation estimate.

**BioM2 1^st^ stage:** 

1. For each pathway, repeat the following steps B times:
  - Draw a cross-validation set from D_k_ with replacement.
  - Fit a model f^1^ to the cross-validation data.
  - Predict the model on the out-of-bag samples.
2. For each block, determine the mean prediction score ${\hat{\mathrm{Y}}}_{kMavg}^{(1)}=\frac{1}{B}{\sum}_{b=1}^B{\hat{\mathrm{Y}}}_{kMb}^{(1)}$.

**BioM2 2^nd^ stage:** 

1. Combine ${\hat{\mathrm{Y}}}_{kMavg}^{(1)}$across all K pathways with ${X}_{pM}$ to generate D^2^.
2. Fit a model f^2^ to D^2^ with cross-validation.
3. Determine the error metric Err_cv_ to evaluate the model performance.

In this framework, the training data D consists of biological measures annotated to K pathways and a training target label indicating the case–control status under exploration. Genome-wide DNA methylation data: 68 MDD patients and 68 healthy controls. Similarly, genome-wide gene expression data include 69 depressive disorder patients and 69 healthy controls. Resampling methods are used to estimate model performance. In stage 1, different supervised learning models (termed as stage-1 models) can be employed to create pathway-level features; subsequently, these pathway-level features and the first-level features not mapped to pathways will be used to construct the stage-2 data. In stage 2, different supervised models (referred to as stage-2 models) also can be applied for prediction purposes. Cross-validation is the primary resampling method employed to estimate model performance. However, other resampling methods [36, 37] can be used as alternatives. Feature selection methods can be incorporated at both stages to identify useful predictors within the resampling procedure, depending on the specific biological contexts or research questions. Note that, in stage-2 data, only positively outcome-associated pathway-level features were used for prediction. The selection of positive pathway-level features was due to the fact that random data can give rise to features inversely associated with the outcome [38].

### Model parameter setting and evaluation metrics

To validate the effectiveness of *BioM2*, we compared it with 10 distinct conventional machine learning models. These models include Decision Trees, Support Vector Machines, Random Forests, Naive Bayes, Logistic Regression, L2-regularized Logistic Regression, k-Nearest Neighbors, Generalized Boosted Models, Gaussian Processes, and Elastic Nets. It should be noted that each of these 10 models can be applied as the base model to both stage 1 and stage 2 of the *BioM2* framework. In this framework, base models are treated as hyperparameters, and the most suitable model is selected through a cross-validation procedure. At the first stage, features with a case–control difference above a predetermined cutoff (measured by the absolute value of the Pearson correlation coefficient (PCC)) are selected for each pathway during each resampling iteration. The stage-1 correlation cutoffs explored include 0, 0.05, 0.1, 0.2, 0.3, and 0.5. In the absence of any feature meeting the threshold, the top 10 most correlated features for each pathway are identified using the PCC. For the unmapped features, the top (0, 100, 300, 500, 1000) most significant features were used. At the second stage, we remove highly correlated pathway-level features (stage-2 correlation cutoff exceeding 0.8 or 1.0) to minimize pairwise correlation. Additionally, only those features showing a positive association with the diagnosis were selected for training the second-level models. The hyperparameters were optimized through a rigorous repeated cross-validation process to ensure the robustness and reliability of the *BioM2* framework.

Model performance is quantified using the area under the receiver operating characteristic curve (AUC), the precision–recall area under the curve (PRAUC), balanced accuracy (BAC), and PCC, comparing predicted values against actual values. The significance of pathway-level features in contributing to classification is assessed using the *P* value and PCC, facilitating the ranking of feature importance.

### Comparative machine learning methods

This comparison was designed to assess the predictive performance of 10 distinct conventional machine learning methods in relation to *BioM2.* We implemented feature selection to enhance the predictive accuracy of conventional machine learning methods and to streamline the variable set. The top (500, 1000, 2000, 5000, 10 000, 15 000) most significant features were identified using PCC to create varying sets of relevant features. The optimal feature set for each method was determined through a rigorous process of 10 times repeated five-fold cross-validation, utilizing the AUC as the primary performance metric. The feature set yielding the best AUC was then selected for the final performance evaluation. It is worth noting that we did not perform hyper-parameter optimization on either *BioM2* or the conventional machine learning methods to maintain uniformity in comparison. The machine learning models for both *BioM2*’s stage 1 and stage 2, as well as conventional methods, are implemented using the *mlr3* R package [39].

Gene set enrichment analysis (GSEA) [40] was performed to further contrast the functional output with that derived from *BioM2*. A list of ranked CpGs or genes is required as input for GSEA; we, therefore, ranked all CpGs or genes by their Pearson’s correlation scores for the enrichment analysis by using the *methylGSA* and *fgsea* Bioconductor R package [41, 42], adhering to prespecified parameter settings, specifically selecting pathways containing between 20 and 200 genes. To underscore the enrichment results, the *P* values and the enrichment scores were reported.

### Identification of pathway-level modules

Pathway-level features generated by the stage 1 of *BioM2* deserve further analysis. To elucidate processes potentially relevant to illness, we employed the WGCNA technique. This approach clusters pathway-level features exhibiting similar profiles into distinct groups, referred to as pathway modules, thereby facilitating the identification of shared biological functions. As described below, according to GO pathway ancestral relationship and *P* value, differential pathway modules with high biological explanatory fraction were identified to explore potential biological patterns linked to disease. For this analysis, GO pathway ancestry relationships were obtained from the *GO.db* and *AnnotationDbi* R package [43]. The detailed procedure was as follows:

(1) Clustering of pathway-level features into pathway modules in healthy individuals using a WGCNA approach. (2) Calculating the eigenvectors of the pathway modules for the full sample using singular value decomposition (SVD). (3) *P* values, quantifying the association between each module eigenvector and the phenotype (healthy/patient) were calculated using Wilcoxon tests. (4) Calculating the biological explanatory fraction for each pathway module. Biological explanatory fraction: the proportion of GO pathways in a given module that trace back to the same ancestral GO pathway, relative to the total number of pathways in that module. This calculation takes into account the hierarchical structure of GO pathways, where each pathway can have several ancestors within the GO taxonomy. (5) Based on the *P* value and biological explanatory fraction, we selected the illness-relevant modules with high biological interpretability.

### Computational considerations

The *BioM2* package was incorporated into CRAN, but due to the size limit of external data and the time limit during the CRAN check, an exemplary dataset with 20 subjects from genome-wide DNA methylation dataset and 20 subjects from genome-wide gene expression data is provided in the tutorial for demonstration purposes. This dataset can be accessed after installing the package. The full datasets investigated in the present study are available from the GEO database (GSE198904 and GSE46743). We have implemented the parallel computing strategy using the *parallel* R package.

## Results

### Prediction performance comparison

The prediction performance of *BioM2* with GO metadata and 10 different traditional machine learning methods on the both datasets was evaluated using four metrics, AUC, PCC, BAC, and PRAUC, along with their respective SDs in 10 repetitions of five-fold cross-validation (Tables 1 and 2). For DNA methylation data (Table 1), among the 10 conventional machine learning methods, while the L2-regularized logistic regression model outperformed *BioM2* in the BAC metric, and both L2-regularized logistic regression and support vector machine models outperformed *BioM2* in the PCC metric, *BioM2* demonstrated superior performance in the metrics of AUC and PRAUC. For gene expression data (Table 2), Naive Bayes model outperformed *BioM2* in the metric of BAC. Nevertheless, the *BioM2* model showed superior performance compared to 10 other conventional models in three metrics of AUC, PCC, and PRAUC. The detailed prediction performance for the *BioM2* base model is shown in Supplementary TableS1 and 2 for DNA methylation and gene expression data, respectively.

**Table 1:** Prediction performance comparison for genome-wide DNA methylation data between *BioM2* with pathway-stratified and conventional machine learning

Model	AUC, mean ± SD	PCC, mean ± SD	BAC, mean ± SD	PRAUC, mean ± SD
*BioM2* (GO_BP)	**0.932 ± 0.015**	0.745 ± 0.025	0.865 ± 0.022	**0.942 ± 0.015**
Decision Tree	0.685 ± 0.068	0.339 ± 0.128	0.663 ± 0.065	0.681 ± 0.070
Support Vector Machine	0.926 ± 0.015	0.756 ± 0.031	0.844 ± 0.026	0.931 ± 0.018
Random Forest	0.931 ± 0.016	0.730 ± 0.025	0.862 ± 0.021	0.938 ± 0.016
Naive Bayes Model	0.893 ± 0.021	0.711 ± 0.061	0.847 ± 0.031	0.867 ± 0.026
Logistic Regression	0.535 ± 0.037	0.059 ± 0.076	0.529 ± 0.036	0.536 ± 0.029
L2-regularized Logistic Regression	0.928 ± 0.013	**0.751 ± 0.026**	**0.865 ± 0.019**	0.939 ± 0.014
k-Nearest Neighbor	0.889 ± 0.029	0.668 ± 0.039	0.667 ± 0.018	0.916 ± 0.021
Generalized Boosted Model	0.890 ± 0.022	0.682 ± 0.040	0.805 ± 0.022	0.891 ± 0.019
Gaussian Process	0.913 ± 0.018	0.707 ± 0.027	0.838 ± 0.031	0.923 ± 0.019
Elastic Net	0.859 ± 0.025	0.624 ± 0.040	0.785 ± 0.020	0.877 ± 0.027

*Notes*: The table presents the AUC, PCC, BAC, and PRAUC values along with their corresponding SDs for the respective classification tasks, based on 10 repetitions of five-fold cross-validation. *BioM2* (GO_BP) refers to the *BioM2* model utilizing gene ontology pathways specifically related to biological processes. The *BioM2* base model used is “L2-regularized logistic regression” with the following settings: stage-1 correlation cutoff at 0.3, top 300 unmapped features, and stage-2 correlation cutoff at 0.8.

**Table 2:** Prediction performance comparison for genome-wide gene expression data between *BioM2* with pathway-stratified and conventional machine learning

Model	AUC, mean ± SD	PCC, mean ± SD	BAC, mean ± SD	PRAUC, mean ± SD
*BioM2* (GO_BP)	***0.628*** ± **0.026**	***0.218*** ± **0.044**	0.595 ± 0.015	***0.648*** ** ±** **0.032**
Decision Tree	0.541 ± 0.051	0.061 ± 0.101	0.527 ± 0.051	0.542 ± 0.038
Support Vector Machine	0.614 ± 0.019	0.200 ± 0.040	0.592 ± 0.030	0.623 ± 0.026
Random Forest	0.597 ± 0.026	0.161 ± 0.048	0.572 ± 0.019	0.607 ± 0.028
Naive Bayes Model	0.607 ± 0.024	0.204 ± 0.045	**0.599** ± **0.021**	0.581 ± 0.021
Logistic Regression	0.535 ± 0.046	0.055 ± 0.083	0.526 ± 0.040	0.534 ± 0.037
L2-regularized Logistic Regression	0.526 ± 0.023	0.044 ± 0.042	0.510 ± 0.025	0.545 ± 0.028
k-Nearest Neighbor	0.553 ± 0.052	0.097 ± 0.084	0.542 ± 0.043	0.562 ± 0.042
Generalized Boosted Model	0.554 ± 0.042	0.085 ± 0.071	0.537 ± 0.029	0.569 ± 0.034
Gaussian Process	0.627 ± 0.019	0.199 ± 0.041	0.575 ± 0.022	0.629 ± 0.021
Elastic Net	0.494 ± 0.030	NA	0.494 ± 0.026	0.520 ± 0.023

*Notes*: The table presents the AUC, PCC, BAC, and PRAUC values along with their corresponding SDs for the respective classification tasks, based on 10 repetitions of five-fold cross-validation. *BioM2* (GO_BP) refers to the *BioM2* model utilizing gene ontology pathways specifically related to biological processes. The *BioM2* base model used is “Random Forest” with the following settings: stage-1 correlation cutoff at 0.2, top 300 unmapped features, and stage-2 correlation cutoff at 1.0.

### Properties of top-ranked pathway-level features

**Figure 2:**
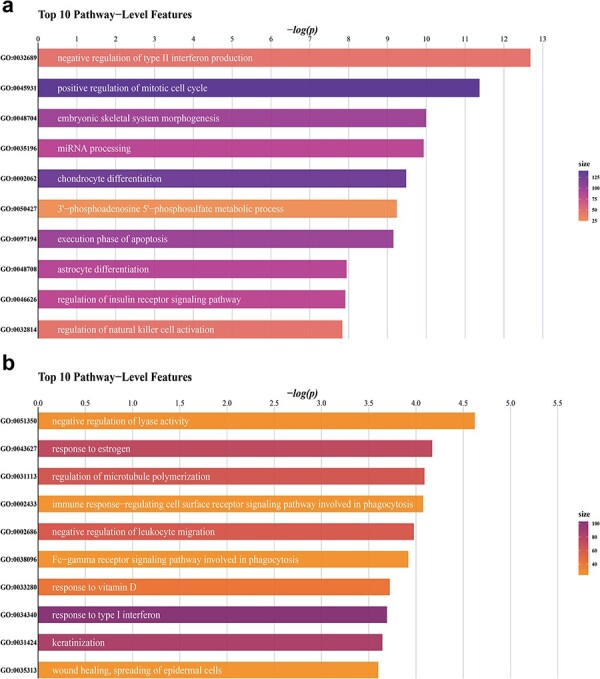
Top-ranked GO pathway-level features. a.) based on genome-wide DNA methylation data. b.) based on genome-wide gene expression data. The top ten outcome-associated gene ontological pathway-level features are illustrated and ranked according to the negative log *P* value, respectively. *BioM2* was applied to generate the pathway-level features. Colors indicate the dimensionality of the data underlying a given pathway-level feature (i.e., ``size'' reflects the number of genes within a given pathway).

**Figure 3:**
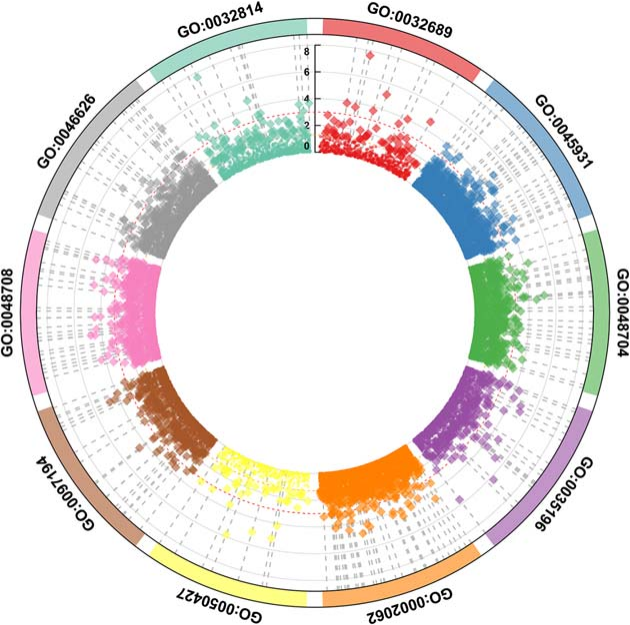
Top-ranked GO pathways and the significance of individual input features in the respective pathways based on genome-wide DNA methylation data. *BioM2* was applied to generate the pathway-level features. The negative log *P* value for the association of input features with the outcome was computed using logistic regression. The same pathway is drawn in the same color. The dots or diamonds present the significance of individual CpGs within these pathways. Significant CpGs are denoted as filled diamonds above the dashed red line representing the nominal significant *P* value of .05.

**Figure 4:**
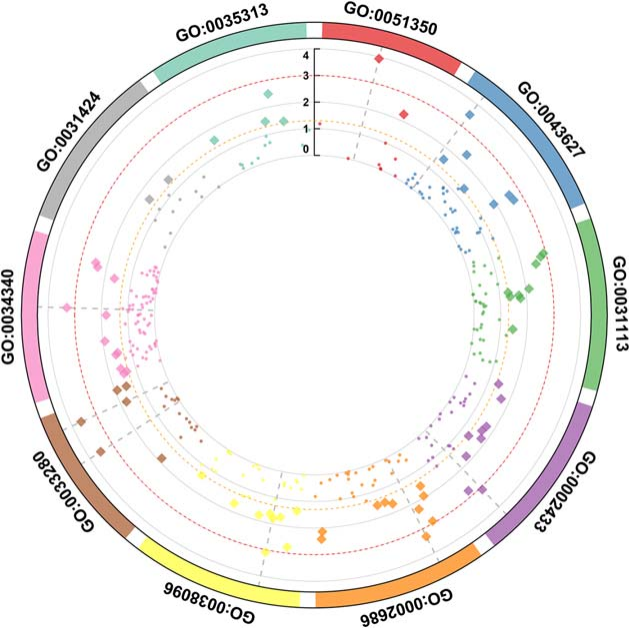
Top-ranked GO pathways and the significance of individual input features in the respective pathways based on genome-wide gene expression data. *BioM2* was applied to generate the pathway-level features. The negative log *P* value for the association of input features with the outcome was computed using logistic regression. The same pathway is drawn in the same color. The dots or diamonds present the significance of individual CpGs within these pathways. Significant CpGs are denoted as filled diamonds above the dashed red line representing the nominal significant *P* value of .05.

Figure 2a shows the 10 most important gene ontological pathway-level features, colored according to the dimensionality of the underlying DNA methylation data (Fig. 2b for gene expression data). The statistical metrics for each of these pathways derived from DNA methylation and gene expression data are shown in Supplementary Tables 3 and 4. Figures 3 and 4 illustrate the significance of individual CpGs (DNA methylation data) or genes (gene expression data) falling into the respective gene ontological pathways. Supplementary Figs. 1 and 2 show the outcomes of a network analysis focusing on the top 10 pathway-level features. The DNA methylation analysis highlighted significant associations of astrocyte differentiation, mitotic cell cycle and type II interferon production pathways with additional pathways. Conversely, the gene expression analysis demonstrated more pronounced associations with the response to vitamin D and the negative regulation of leukocyte migration pathways, suggesting that distinct biological processes are preferentially engaged across different levels of genomic regulation. Supplementary Fig. 3 illustrates the significance of individual gene ontological pathway-level features derived from the DNA methylation data, categorizing them under their corresponding GO ancestors (see Supplementary Fig. 4 for gene expression data). The findings reveal that significant GO pathway-level features in DNA methylation data predominantly relate to inflammation and immune functions. Similarly, the gene expression data analysis uncovers that the most significant pathways are associated with immune functions. Our subsequent analysis suggests that the size of the pathway did not markedly affect the results in either data modality, indicating the robustness of our findings across different pathway scales (see Supplementary Tables 5 and 6). GSEA results derived from DNA methylation data are shown in Supplementary Table 7 and those derived from gene expression data in Supplementary Table 8. Only 10 most outcome-associated pathways were used for this purpose. The findings demonstrate that the pathways identified by *BioM2* and GSEA showed only minimal overlap, a likely consequence of the methodological differences between the two approaches.

### High-level pathway-based functional modules

**Figure 5:**
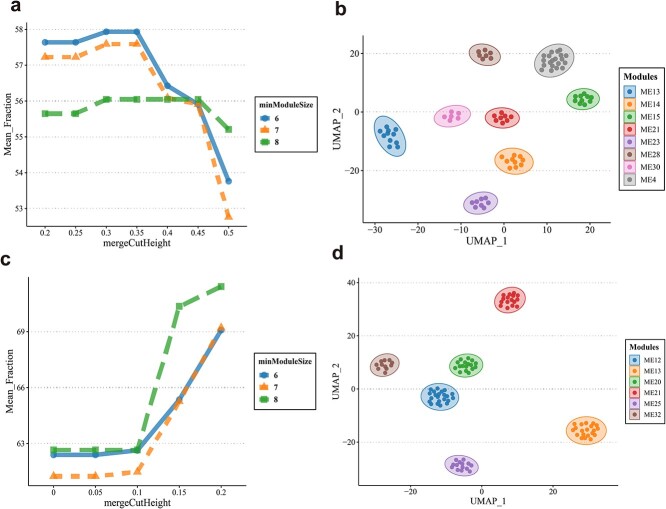
a.) Parameter selection considered for modular clustering with WGCNA for genome-wide DNA methylation data. Clustering with WCGNA requires two parameters minModuleSize, mergeCutHeight. Mean_Fraction is determined by these two parameters, with a higher Mean_Fraction indicating a more biologically explanatory clustering. b.) UMAP shows all the biologically explainable modules for genome-wide DNA methylation data. Each dot represents a pathway-level feature, a module consists of multiple pathway-level features, and pathway features from the same module are represented by the same colour. c.) Parameter selection considered for modular clustering with WGCNA for genome-wide gene expression data. Clustering with WCGNA requires two parameters minModuleSize, mergeCutHeight. Mean_Fraction is determined by these two parameters, with a higher Mean_Fraction indicating a more biologically explanatory clustering. d.) UMAP shows all the biologically explainable pathway modules for genome-wide gene expression data. Each dot represents a pathway-level feature, a module consists of multiple pathway-level features, and pathway features from the same module are represented by the same colour.

**Figure 6:**
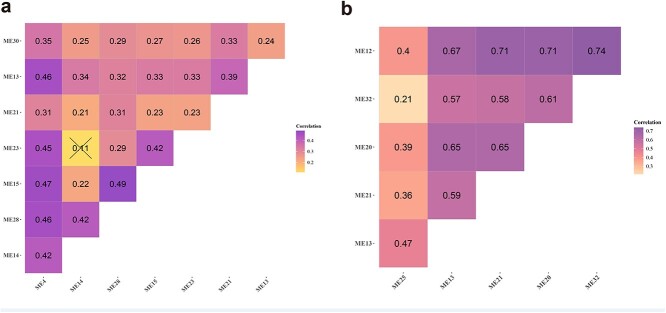
a.) The correlation coefficients between biologically explainable modules derived from genome-wide DNA methylation pathway-level data. b.) The correlation coefficients between biologically explainable modules derived from genome-wide gene expression pathway-level data. The intensity of the shade corresponds to the strength of correlation between the modules, with darker shades indicating a higher correlation. Modules that do not demonstrate a significant relationship are distinctly marked with an `X'.

**Figure 7:**
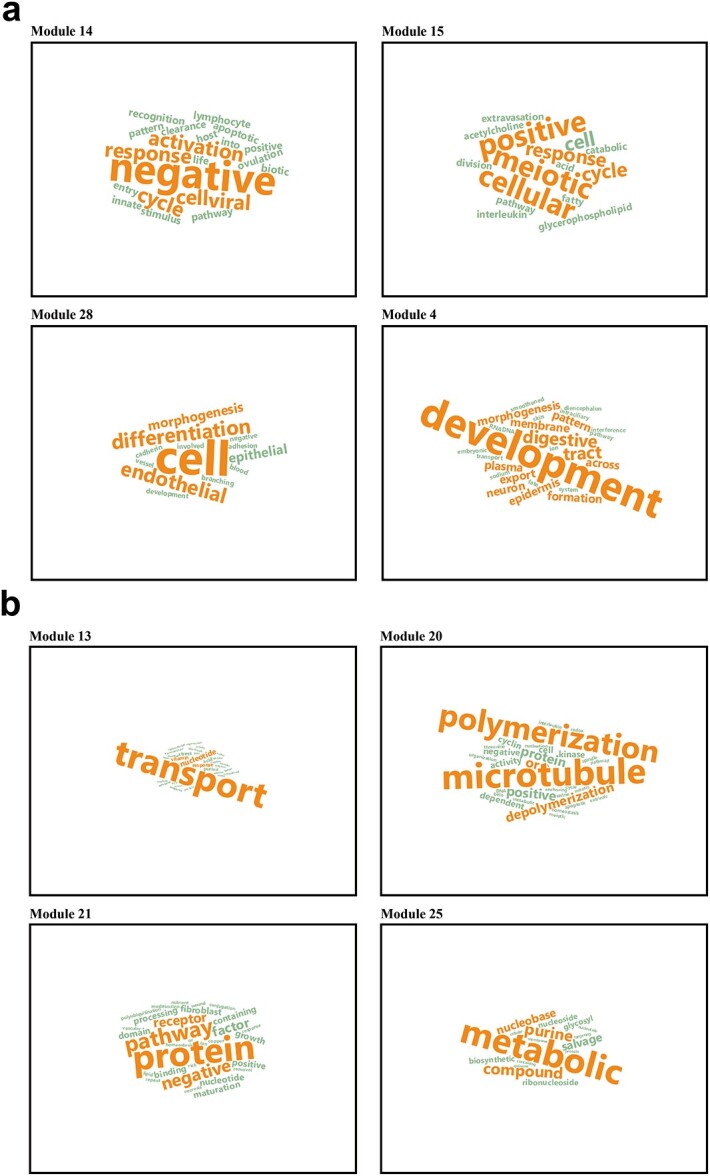
*BioM2* demonstrates the biological semantic information contained in the four most significant modules using Word Cloud a.) for genome-wide DNA methylation data. b.) for genome-wide gene expression data. The frequency of a word's appearance is directly proportional to its size in the visualization – the more frequent the word, the larger it appears. Additionally, the top quarter of most commonly occurring words are distinctively highlighted in orange for emphasis.

**Figure 8:**
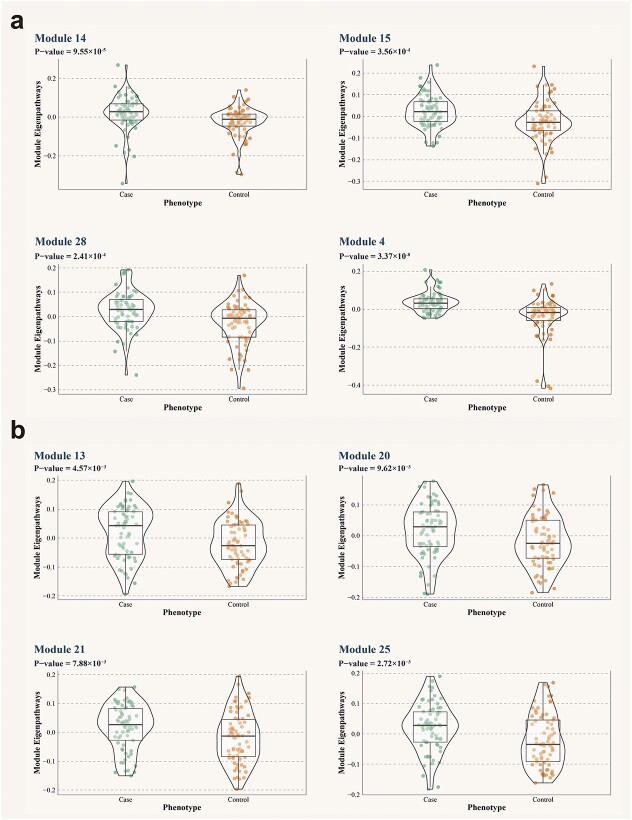
*BioM2* demonstrates the differences in the four most significant modules using violin chart. a.) for genome-wide DNA methylation data. b.) for genome-wide gene expression data. The horizontal coordinates are the phenotypes, indicating the two groups that suffer from depression or not. Vertical coordinates are the eigenvectors that make up the pathway module.

Figure 5a shows that for DNA methylation data, the important parameters minModule and mergeCutHeight for gene ontological pathway-level features clustering are determined by the biological explanatory fraction (Fig. 5c for gene expression data). We identified and visualized pathway-based modules using uniform manifold approximation and projection (UMAP) in Fig. 5b (Fig. 5d for gene expression data). Figure 6a and b describes the correlation among these investigated modules. Figure 7a shows the biological explanatory semantic information contained in each of the four most significant differential pathway modules (Fig. 7b for gene expression data). The significance for each differential pathway module in the case–control analysis, based on DNA methylation data, is visually presented in Fig. 8a (Fig. 8b for gene expression data). The detailed statistical metrics for these differential pathway modules, derived from DNA methylation and gene expression data, are shown in Supplementary Tables 9 and 10, respectively.

## Discussion

Here, we developed *BioM2*, a publicly available software tool designed for enhancing phenotype prediction. This tool integrates biological metadata with machine learning models trained on omics data, aiming to identify biological signals related to specific phenotypes. Through two concise case studies, *BioM2*’s effectiveness was showcased, outperforming traditional, biologically uninformed machine learning approaches in both prediction accuracy and functional insights pertinent to the phenotype in question. While traditional methods like the L2 model exhibited smaller SDs, as shown in Table 1, likely due to their simpler architecture, *BioM2*’s more complex two-stage process may introduce additional variability, resulting in slightly higher SDs. Nonetheless, *BioM2* demonstrated superior overall performance, particularly in terms of AUC and PRAUC. Unlike the previous BioMM framework [23], the updated *BioM2* model integrates unmapped omics features, which could help improve the accuracy of disease risk prediction, as demonstrated in Supplementary Table 11. To mitigate potential overfitting and address imbalances in the DNA methylation dataset, we employed permutation tests and scaled up the control samples to ensure a fair comparison. The results, detailed in Supplementary Tables 12 and 13, confirm the reliability and superior performance of *BioM2* compared to traditional approaches. For gene expression data, we also used the full dataset for prediction due to the relatively balanced sample sizes between cases and controls. The results, as shown in Supplementary Table 14, consistently support the superior performance of *BioM2*.

A key feature of *BioM2* is its ability to pinpoint the most informative signals within an omics-derived signature. It modularizes pathway-level features derived from biological process annotation and incorporates a suite of visualization tools to aid in their interpretation. In our study, *BioM2* was applied to genome-wide DNA methylation and gene expression data, but its capabilities can extend to other omics data types, such as genome-wide association studies and metabolomic data. One of *BioM2*’s strengths is its versatility in integrating biological knowledge. While it can utilize gene ontological categories, it is also capable of incorporating other pathway resources and biological meta-information, including KEGG pathways and co-expression networks. *BioM2* offers flexibility regarding the choice of machine learning algorithms within its framework. Recognizing that the use of supervised methods can significantly increase computational demands, *BioM2* includes efficient parallelization features to address this challenge. Additionally, its modular architecture not only facilitates direct end-to-end prediction but also allows for independent analyses of its two distinct stages.

It is critical to carefully interpret the stage-2 data features identified by *BioM2* as being associated with the desired outcome. This need for caution arises because, within a specified aggregation unit (such as a biological pathway), supervised machine learning selects the most predictive features from stage-1 data. These selected features are then integrated to form a component of the stage-2 data. Such integration might lean heavily on a few stage-1 features strongly associated with the outcome, potentially leading to diminished biological specificity in relation to the aggregation unit. This may be the reason for the results generated on the case study data to differ strongly with those obtained by GSEA, which incorporates the full set of features. Additionally, it is important to recognize that the same stage-1 features might be mapped across multiple aggregation units. This overlap can further dilute the biological specificity of the analysis. Moreover, *BioM2*’s current design, which prioritizes maximizing predictive performance, does not take into account variations in pathway size. Consequently, larger pathways, which are more likely to encompass highly predictive features, may inadvertently skew the pathway rankings. Our *post hoc* analysis partially confirms that pathway size did not significantly influence the findings in either of the two data modalities. These methodological nuances are critical for a nuanced understanding of the pathway-level predictors’ rankings derived from *BioM2*, ensuring that interpretations are both accurate and meaningful.

Among the most significant pathway-level features identified using genome-wide DNA methylation data, the “execution phase of apoptosis” stands out as an inflammation-related biological pathway associated with MDD [44]. Furthermore, there is compelling evidence connecting “astrocyte differentiation” to MDD, as recent studies have demonstrated heightened inflammation of astrocytes among MDD patients [45]. Additionally, untreated MDD has been linked to a reduction in natural killer cells, further underscoring the relevance of the “regulation of natural killer cell activation” pathway to MDD [46]. Our analysis of genome-wide gene expression data has also revealed a significant association between the “negative regulation of leukocyte migration” and MDD [47]. At a higher level of organization, the pathway features are grouped into functional modules. We observed that the most significant module derived from genome-wide DNA methylation data is associated with anatomical structure development, specifically cerebellar cortex development and central nervous system neuron development [48, 49] (referred to as the top module ME4, as detailed in Supplementary Table 7). The significant correlation between the top module ME4 and other functional modules implies complex interrelationships among various biological processes. For instance, modules such as “meiosis and cell cycle regulation” (ME15), “negative regulation of the immune response” (ME13) [50], “regulation of endothelial differentiation” (ME28) [51], and “post-embryonic development” (ME23) may play significant roles in the pathogenesis of MDD. This interconnectivity indicates that perturbations in one pathway could have cascading effects on others, contributing to the complex etiology of MDD. The observed correlations suggest potential pathways for further research to explore these relationships in the context of MDD. In contrast, the module derived from gene expression data is most significantly associated with MDD through the negative regulation of microtubule (highlighted as the top module ME20 as illustrated in Supplementary Table 8). This observation may potentially underscore the relevance of anomalies in microtubule-associated proteins in MDD [52, 53]. Importantly, the integration of methylome and transcriptome data has revealed pathway-based modules linked to the regulation of immune function. This discovery could provide further insights into the possible relationship between depression and immune function [50, 54]. We also prioritized genes derived from *BioM2* based on both DNA methylation data (Supplementary Tables 15 and 16) and gene expression data (Supplementary Tables 17 and 18). Notable genes from the DNA methylation data include PRKCZ and PRKCB, both members of the Protein Kinase C family involved in the MAPK pathway, which may be relevant to MDD [55, 56], and PUM1, which regulates neurogenesis in conjunction with PUM2, playing significant roles in the mammalian nervous system [57]. For gene expression data, the FGF family is implicated in depression and potentially other psychiatric disorders, with FGF23 levels upregulated in depressive patients following lithium treatment [58]. Additionally, PAK1 mRNA levels are altered in the prefrontal cortex and hippocampus of depressed subjects [59], and RT-PCR revealed reduced expression of TGFBR2 in the functional forebrain regions and hippocampus of rodent models of depression [60].

In summary, we described the development of the CRAN R package *BioM2*, which offers a biologically informed machine learning framework for phenotype prediction using either genome-wide DNA methylation data or transcriptome-wide gene expression data, and for exploring potential biological mechanisms linked to the phenotype. We demonstrated its application and predictive accuracy using two example datasets while incorporating biological pathway information via GO pathways. The *BioM2* package supports the ranking and visualization of features based on their contribution to successful prediction. Furthermore, it enables the clustering of pathway-level features with similar patterns into pathway modules and facilitates their analysis and visualization.

**Key Points**

- *BioM2* introduces a novel, biologically informed machine learning framework specifically designed for high-dimensional omics data analysis, effectively bridging a critical gap in current analytical tools.
- Leveraging biological insights, *BioM2* surpasses conventional machine learning models in predictive accuracy, showcasing its advanced analytical capabilities.
- The package’s versatility and efficacy are demonstrated across various omics datasets, including genome-wide DNA methylation and transcriptome-wide gene expression, underscoring its wide-ranging research applications.
- A distinctive feature of *BioM2* is its ranking of predictor variables by biological categories, like GO pathways, facilitating result interpretability and enabling modular network analysis to explore the systems-level biology driving predictions.

## Supplementary Material

BIoM2_zhang_suppl_Figures

BioM2_suppl_final_v1_bbae384

Supplementary_Table_10_gene_expression_module_bbae384

Supplementary_Table_9_DNA_methylation_module_bbae384

## Data Availability

All analyses utilized publicly available data from the GEO database (GSE198904 and GSE46743). The BioM2 software package, developed in R, is accessible on CRAN, featuring example datasets and comprehensive documentation. Data and code would be available upon request. Additionally, we offer a detailed set of online tutorials available on GitHub (https://github.com/BioTransAI/BioM2), facilitating user understanding and application of BioM2.
