# Supplementary material for: BioM2: biologically informed multi-stage machine learning for phenotype prediction using omics data
Source: Brief Bioinform. 2024 Aug 10;25(5):bbae384. doi: 10.1093/bib/bbae384 (PMC11316398; doi:10.1093/bib/bbae384)

**Supplementary Materials**

**Supplementary Figure 1. Internal correlations between top-ranked GO pathway-level features based on genome-wide DNA methylation data.** Dots belonging to the same pathway are depicted in identical colors. Solid lines link correlations among features within pathways, while dashed lines connect correlations between pathways identified in the top quartile of strongest relationships.


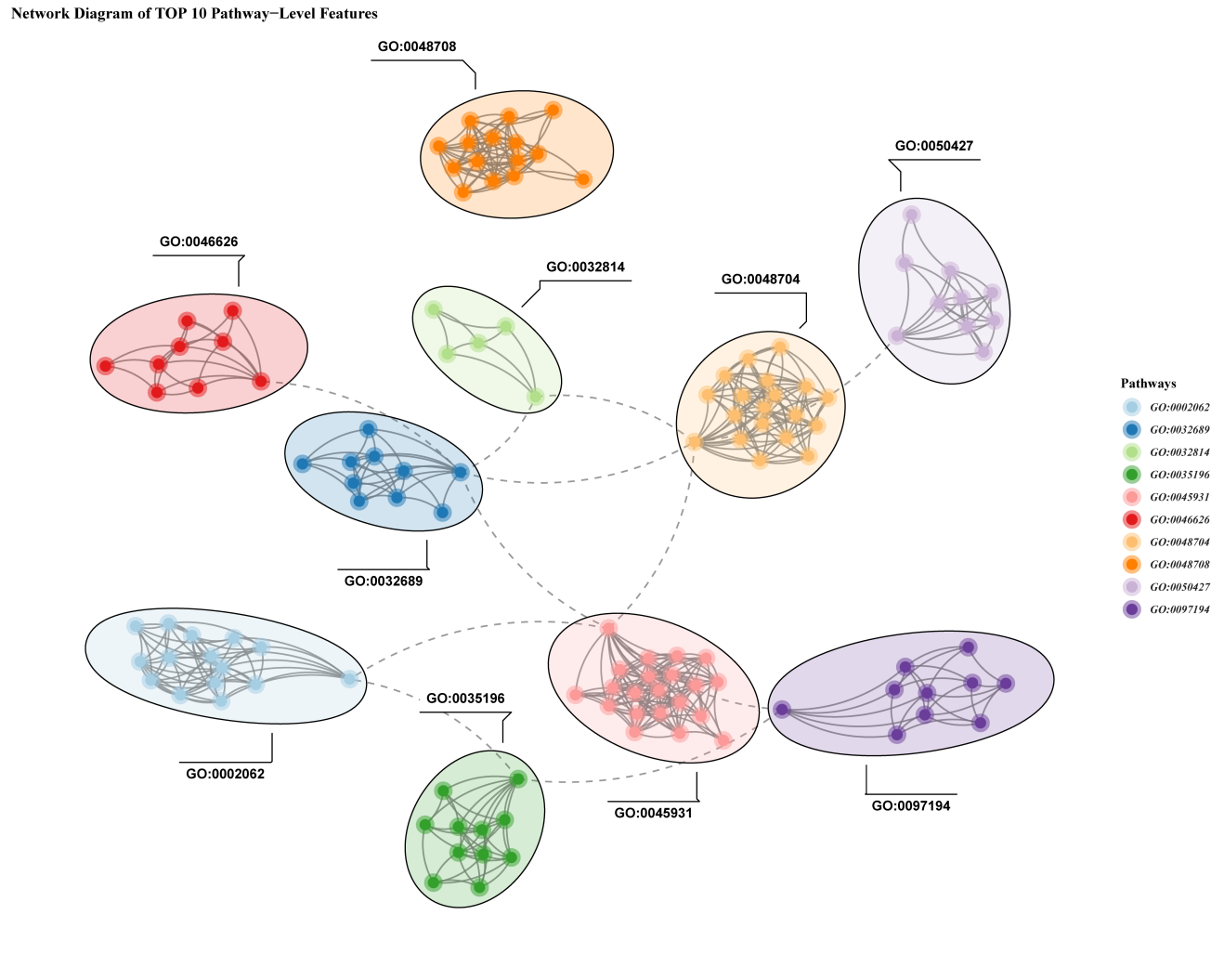


**Supplementary Figure 2. Internal correlations between top-ranked GO pathway-level features based on genome-wide gene expression data.** Dots belonging to the same pathway are depicted in identical colors. Solid lines link correlations among features within pathways, while dashed lines connect correlations between pathways identified in the top quartile of strongest relationships.


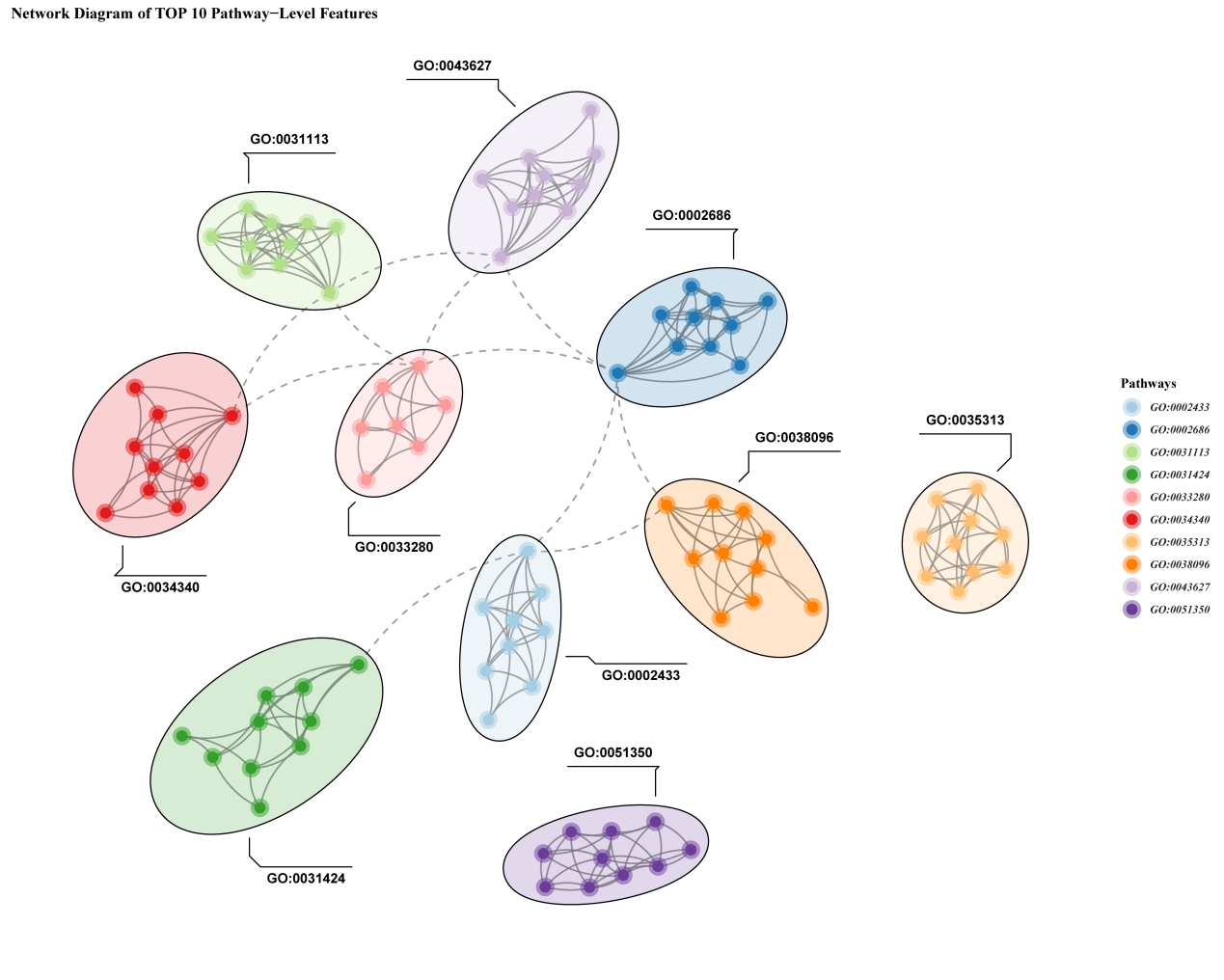


**Supplementary Figure 3. Top eight GO Ancestors containing the most GO pathways and the significance of individual pathway-level features in the respective GO Ancestors based on genome-wide DNA methylation data.** BioM2 was applied to generate the pathway-level features. GO ancestral relationship from GO.db package.The negative log P value for the association of pathway-level features with the phenotype was computed using Wilcoxon tests. The same GO ancestor is drawn in the same color. The dots present the significance of individual GO pathways within these ancestors. Significant GO pathways are denoted as filled dots above the dashed orange line.


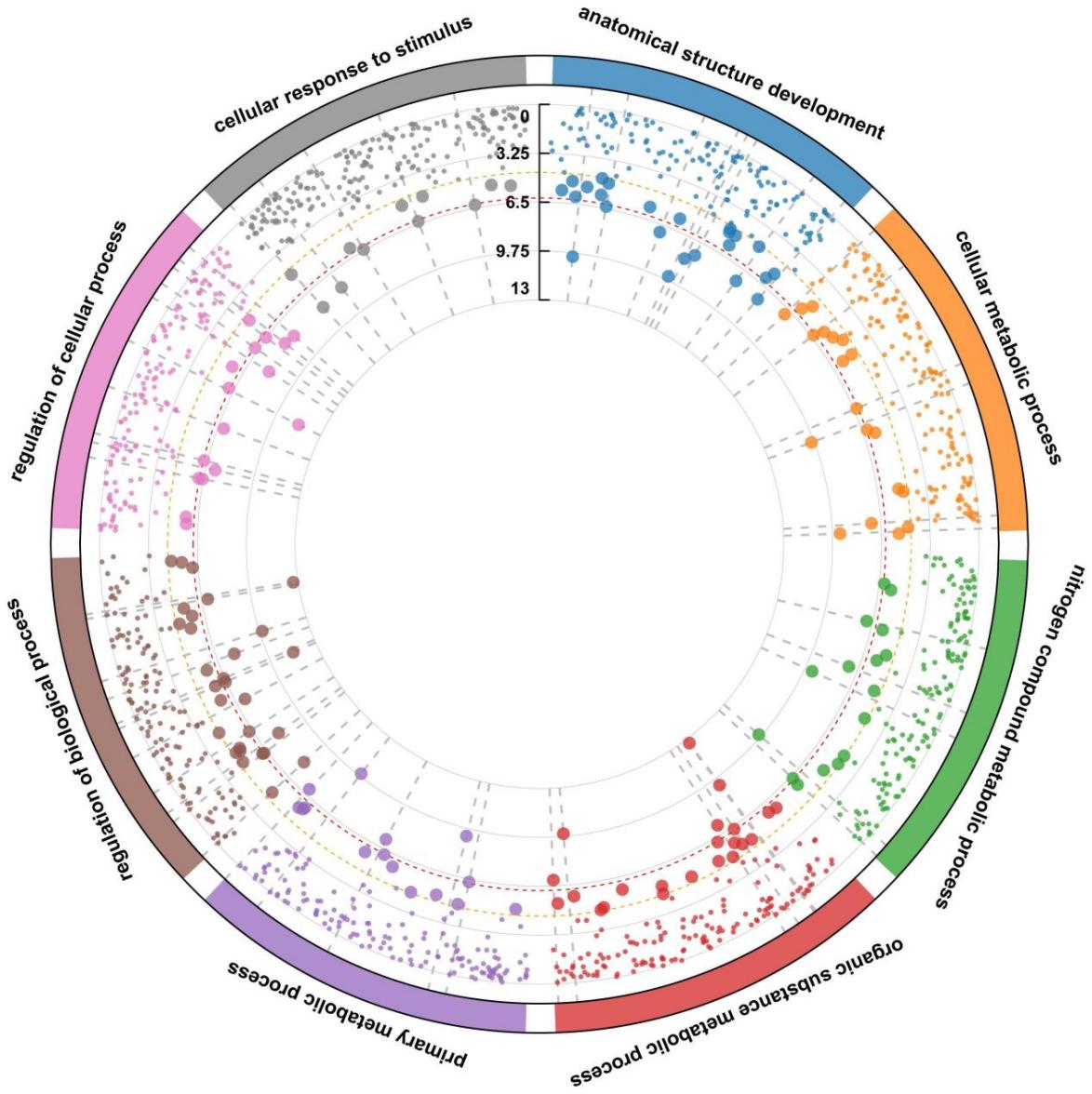


**Supplementary Figure 4. Top eight GO Ancestors containing the most GO pathways and the significance of individual pathway-level features in the respective GO Ancestors based on genome-wide gene expression data.** BioM2 was applied to generate the pathway-level features. GO ancestral relationship from GO.db package.The negative log P value for the association of pathway-level features with the phenotype was computed using Wilcoxon tests. The same GO ancestor is drawn in the same color. The dots present the significance of individual GO pathways within these ancestors. Significant GO pathways are denoted as filled dots above the dashed orange line.


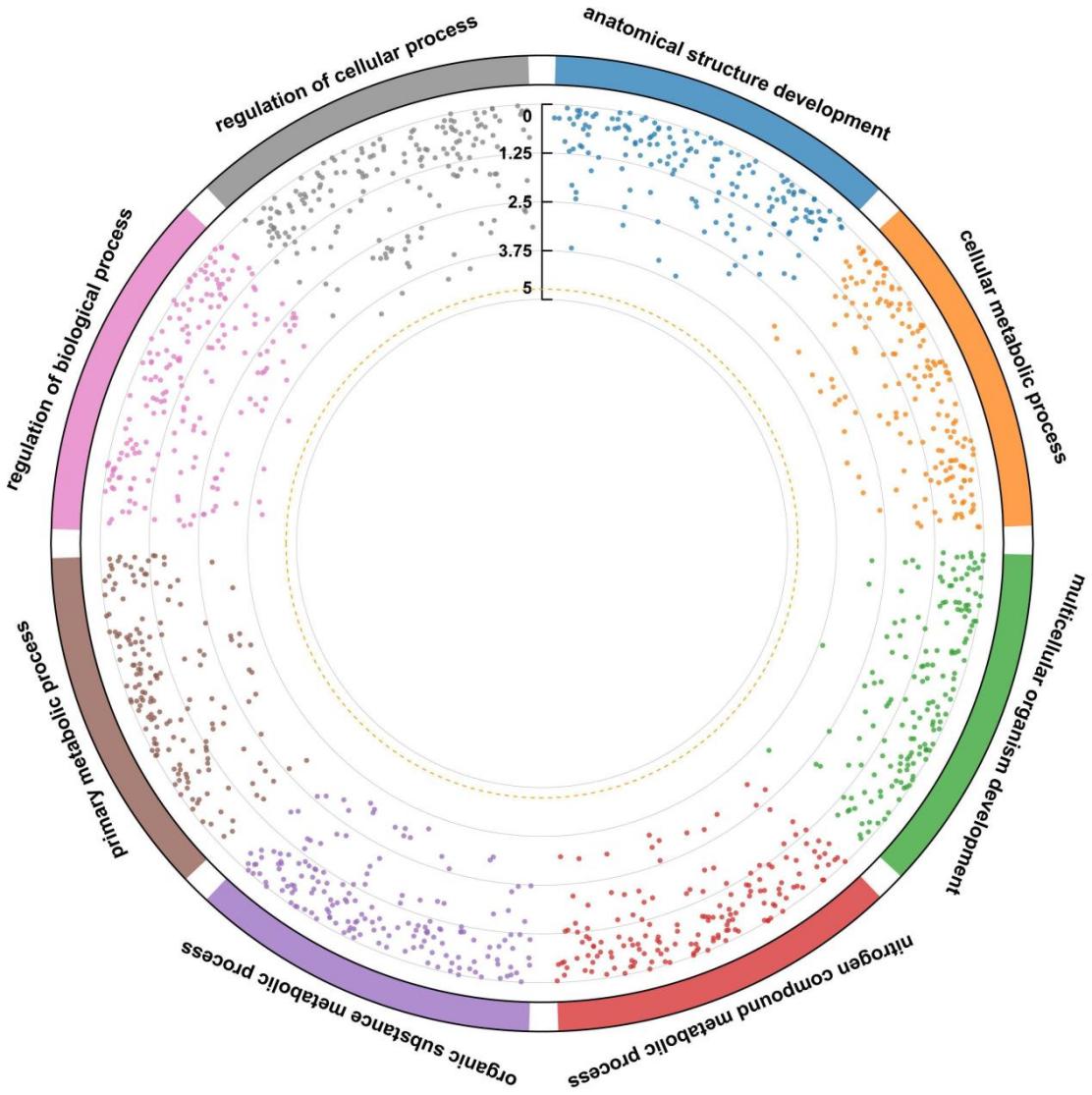

Supplement: BIoM2_zhang_suppl_Figures [file biom2_zhang_suppl_figures.docx]
