# Supplementary material for: BioM2: biologically informed multi-stage machine learning for phenotype prediction using omics data
Source: Brief Bioinform. 2024 Aug 10;25(5):bbae384. doi: 10.1093/bib/bbae384 (PMC11316398; doi:10.1093/bib/bbae384)
Supplement: BioM2_suppl_final_v1_bbae384 [file biom2_suppl_final_v1_bbae384.docx]

**Supplementary Materials**

**Supplementary Table 1.Prediction Performance of BioM2 with Different Base Models on Genome-Wide DNA Methylation Data During Cross-Validation.**

| **BioM2_baseModel** | **AUC** | **PCCs** | | **BAC** | | **PRAUC** |
| --- | --- | --- | --- | --- | --- | --- |
| Decision Tree | 0.596 | 0.169 | 0.580 | | 0.582 | |
| Elastic Net | 0.762 | 0.439 | 0.694 | | 0.781 | |
| Gaussian Process | 0.858 | 0.605 | 0.774 | | 0.873 | |
| Generalized Boosted Model | 0.813 | 0.549 | 0.738 | | 0.827 | |
| k-Nearest Neighbor | 0.857 | 0.607 | 0.685 | | 0.883 | |
| ***L2-regularized Logistic Regression*** | ***0.932*** | ***0.745*** | ***0.865*** | | ***0.942*** | |
| Logistic Regression | 0.511 | 0.051 | 0.525 | | 0.515 | |
| Naive Bayes Model | 0.813 | 0.589 | 0.788 | | 0.771 | |
| Random Forest | 0.911 | 0.690 | 0.836 | | 0.918 | |
| Support Vector Machine | 0.891 | 0.688 | 0.811 | | 0.904 | |

**Supplementary Table 2. Prediction Performance of BioM2 with Different Base Models on Genome-Wide Gene Expression Data During Cross-Validation.**

| **BioM2_baseModel** | **AUC** | **PCCs** | **BAC** | | **PRAUC** |
| --- | --- | --- | --- | --- | --- |
| Decision Tree | 0.527 | 0.053 | 0.527 | 0.524 | |
| Elastic Net | 0.595 | 0.169 | 0.571 | 0.605 | |
| Gaussian Process | 0.612 | 0.201 | 0.583 | 0.613 | |
| Generalized Boosted Model | 0.620 | 0.218 | 0.593 | 0.617 | |
| k-Nearest Neighbor | 0.599 | 0.174 | 0.576 | 0.585 | |
| L2-regularized Logistic Regression | 0.578 | 0.132 | 0.554 | 0.587 | |
| Logistic Regression | 0.525 | 0.034 | 0.517 | 0.526 | |
| Naive Bayes Model | 0.605 | 0.197 | 0.596 | 0.581 | |
| ***Random Forest*** | ***0.628*** | ***0.218*** | ***0.595*** | ***0.648*** | |
| Support Vector Machine | 0.555 | NA | 0.501 | 0.558 | |

**Supplementary Table 3. The 10 most important pathways by BioM2 are based on genome-wide DNA methylation data.** Correlation of each pathway-level features with phenotype and the adjusted P-value .

| **ID** | **Description** | **Correlation** | **Pvalue** | **P-value (FDR)** |
| --- | --- | --- | --- | --- |
| GO:0032689 | negative regulation of type II interferon production | 0.576 | 2.067×10^-13^ | 3.482×10^-10^ |
| GO:0045931 | positive regulation of mitotic cell cycle | 0.549 | 4.257×10^-12^ | 3.584×10^-9^ |
| GO:0048704 | embryonic skeletal system morphogenesis | 0.518 | 1.012×10^-10^ | 4.939×10^-8^ |
| GO:0035196 | miRNA processing | 0.516 | 1.173×10^-10^ | 4.939×10^-8^ |
| GO:0002062 | chondrocyte differentiation | 0.506 | 3.294×10^-10^ | 1.109×10^-7^ |
| GO:0050427 | 3'-phosphoadenosine 5'-phosphosulfate metabolic process | 0.500 | 5.718×10^-10^ | 1.604×10^-7^ |
| GO:0097194 | execution phase of apoptosis | 0.497 | 7.039×10^-10^ | 1.693×10^-7^ |
| GO:0048708 | astrocyte differentiation | 0.465 | 1.126×10^-8^ | 2.280×10^-6^ |
| GO:0046626 | regulation of insulin receptor signaling pathway | 0.464 | 1.219×10^-8^ | 2.280×10^-6^ |
| GO:0032814 | regulation of natural killer cell activation | 0.462 | 1.451×10^-8^ | 2.445×10^-6^ |

**Supplementary Table 4. The 10 most important pathways by BioM2 are based on genome-wide gene expression data.** Correlation of each pathway-level features with phenotype and the adjusted P-value.

| **ID** | **Description** | **Correlation** | **Pvalue** | **P-value (FDR)** |
| --- | --- | --- | --- | --- |
| GO:0051350 | negative regulation of lyase activity | 0.351 | 2.382×10^-5^ | 0.0553 |
| GO:0043627 | response to estrogen | 0.333 | 6.728×10^-5^ | 0.0553 |
| GO:0031113 | regulation of microtubule polymerization | 0.329 | 8.117×10^-5^ | 0.0553 |
| GO:0002433 | immune response-regulating cell surface receptor signaling pathway involved in phagocytosis | 0.328 | 8.398×10^-5^ | 0.0553 |
| GO:0002686 | negative regulation of leukocyte migration | 0.324 | 1.051×10^-4^ | 0.0553 |
| GO:0038096 | Fc-gamma receptor signaling pathway involved in phagocytosis | 0.322 | 1.204×10^-4^ | 0.0553 |
| GO:0033280 | response to vitamin D | 0.313 | 1.894×10^-4^ | 0.0675 |
| GO:0034340 | response to type I interferon | 0.311 | 2.030×10^-4^ | 0.0675 |
| GO:0031424 | keratinization | 0.309 | 2.263×10^-4^ | 0.0675 |
| GO:0035313 | wound healing, spreading of epidermal cells | 0.307 | 2.508×10^-4^ | 0.0675 |

**Supplementary Table 5. Effect of pathway size on the correlation of pathway-level features with phenotype (genome-wide DNA methylation data).** *Top_num* means the number of pathway features most relevant to the phenotype.

| **Top_num** | **Pvalue** | **Correlation** |
| --- | --- | --- |
| 10 | 6.774×10^-1^ | 0.150 |
| 20 | 7.162×10^-1^ | 0.086 |
| 50 | 4.101×10^-1^ | 0.119 |
| 100 | 7.432×10^-1^ | 0.033 |
| 500 | 3.902×10^-3^ | 0.128 |
| 1000 | 8.755×10^-7^ | 0.154 |
| 1663 | 1.920×10^-17^ | 0.206 |

**Supplementary Table 6. Effect of pathway size on the correlation of pathway-level features with phenotype (genome-wide gene expression data) .** Top_num means the number of pathway features most relevant to the phenotype.

| **Top_num** | **Pvalue** | **Correlation** |
| --- | --- | --- |
| 10 | 0.419 | -0.288 |
| 20 | 0.622 | -0.117 |
| 50 | 0.462 | -0.106 |
| 100 | 0.560 | -0.058 |
| 500 | 0.205 | -0.056 |
| 1000 | 0.256 | -0.035 |
| 2712 | 0.957 | 0.001 |

**Supplementary Table 7. The 10 most significant pathways by GSEA are based on genome-wide DNA methylation data.** GSEA of DNA methylation data using R package methylGSA.

| **ID** | **Description** | **Pvalue** | **Size** | **Count** |
| --- | --- | --- | --- | --- |
| GO:0007156 | homophilic cell adhesion via plasma membrane adhesion molecules | 1.249×10^-5^ | 177 | 44 |
| GO:0035265 | organ growth | 1.164×10^-3^ | 181 | 42 |
| GO:0023019 | signal transduction involved in regulation of gene expression | 2.329×10^-3^ | 23 | 9 |
| GO:0042133 | neurotransmitter metabolic process | 3.187×10^-3^ | 30 | 10 |
| GO:0010718 | positive regulation of epithelial to mesenchymal transition | 3.522×10^-3^ | 64 | 17 |
| GO:0043171 | peptide catabolic process | 3.865×10^-3^ | 33 | 10 |
| GO:0060706 | cell differentiation involved in embryonic placenta development | 4.292×10^-3^ | 25 | 9 |
| GO:0006024 | glycosaminoglycan biosynthetic process | 4.399×10^-3^ | 128 | 22 |
| GO:0046621 | negative regulation of organ growth | 5.213×10^-3^ | 40 | 12 |
| GO:0055022 | negative regulation of cardiac muscle tissue growth | 5.296×10^-3^ | 30 | 10 |

**Supplementary Table 8. The 10 most significant pathways by GSEA are based on genome-wide gene expression data.** GSEA of genome-wide gene expression data using R package fgsea.

| **ID** | **Description** | **Pvalue** | **Size** | **ES** |
| --- | --- | --- | --- | --- |
| GO:0031349 | positive regulation of defense response | 1.408×10^-10^ | 185 | 0.463 |
| GO:0032612 | interleukin-1 production | 9.285×10^-10^ | 78 | 0.591 |
| GO:0032652 | regulation of interleukin-1 production | 9.285×10^-10^ | 78 | 0.591 |
| GO:0032731 | positive regulation of interleukin-1 beta production | 3.393×10^-9^ | 46 | 0.684 |
| GO:0050729 | positive regulation of inflammatory response | 3.508×10^-9^ | 89 | 0.562 |
| GO:0032635 | interleukin-6 production | 5.021×10^-9^ | 110 | 0.517 |
| GO:0032675 | regulation of interleukin-6 production | 5.021×10^-9^ | 110 | 0.517 |
| GO:0045088 | regulation of innate immune response | 6.8305×10^-9^ | 171 | 0.445 |
| GO:0032732 | positive regulation of interleukin-1 production | 7.066×10^-9^ | 53 | 0.649 |
| GO:0060326 | cell chemotaxis | 7.587×10^-9^ | 166 | 0.444 |

**Supplementary Table 9. Biologically explainable modules by BioM2 are based on genome-wide DNA methylation data.** Modules with a biological explanatory fraction greater than 70 and the adjusted p-values of less than 0.05 were selected.

| **Modules** | **Description** | **Number of pathways** | **Fraction** | **Pvalue** | **Pvalue (FDR)** | **Correlation** |
| --- | --- | --- | --- | --- | --- | --- |
| ME4 | anatomical structure development (brain etc.) | 20 | 70.00 | 3.372×10^-8^ | 5.564×10^-7^ | 0.418 |
| ME14 | negative regulation of virus-associated immune responses | 10 | 70.00 | 9.545×10^-5^ | 3.150×10^-4^ | 0.256 |
| ME28 | regulation of endothelial differentiation | 7 | 85.71 | 2.414×10^-4^ | 7.242×10^-4^ | 0.318 |
| ME15 | meiosis and cell cycle regulation | 10 | 70.00 | 3.555×10^-4^ | 9.776×10^-4^ | 0.282 |
| ME23 | post-embryonic development | 8 | 87.50 | 3.146×10^-3^ | 5.768×10^-3^ | 0.229 |
| ME21 | amino acid transport | 8 | 100.00 | 5.308×10^-3^ | 9.220×10^-3^ | 0.216 |
| ME13 | negative regulation of the immune response | 11 | 81.81 | 1.488×10^-2^ | 1.965×10^-2^ | 0.205 |
| ME30 | negative regulation of biological processes | 6 | 83.33 | 1.599×10^-2^ | 2.030×10^-2^ | 0.213 |

**Supplementary Table 10. Biologically explainable modules by BioM2 are based on genome-wide gene expression data.** Modules with a biological explanatory fraction greater than 70 and the p-values of less than 0.05 were selected.

| **Modules** | **Description** | **Number of pathways** | **Fraction** | **Pvalue** | **Pvalue (FDR)** | **Correlation** |
| --- | --- | --- | --- | --- | --- | --- |
| ME25 | nucleoside metabolic process | 14 | 85.71 | 2.719×10^-3^ | 0.055 | 0.249 |
| ME13 | Multi-biomolecular transport | 20 | 70.00 | 4.568×10^-3^ | 0.062 | 0.231 |
| ME21 | regulation of protein processing | 16 | 81.25 | 7.8800×10^-3^ | 0.064 | 0.214 |
| ME20 | regulation of microtubule | 17 | 70.59 | 9.624×10^-3^ | 0.065 | 0.215 |
| ME32 | T cell differentiation and regulation | 11 | 81.82 | 2.622×10^-2^ | 0.097 | 0.184 |
| ME12 | Leukocytes, T-cells and neutrophil (immunocyte) migration | 22 | 77.27 | 3.0525×10^-2^ | 0.062 | 0.162 |

**Supplementary Table 11. Performance Comparison Between Pathway-Only Model and Pathway + Unmapped Model Using BioM2 Framework for DNA Methylation and Gene Expression Data**

Notes: In the Pathway + Unmapped model, 300 additional unmapped features were selected based on cross-validation for both DNA methylation and gene expression data.

| **Data Modality** | **Stage-2 Feature** | **AUC** | **PCCs** | **BAC** | **PRAUC** |
| --- | --- | --- | --- | --- | --- |
| DNA methylation | Pathway-only | 0.921 | 0.713 | 0.849 | 0.936 |
|  | Pathway + Unmapped | 0.932 | 0.745 | 0.865 | 0.942 |
| Gene expression | Pathway-only | 0.624 | 0.216 | 0.593 | 0.642 |
|  | Pathway + Unmapped | 0.628 | 0.218 | 0.595 | 0.648 |

**Supplementary Table 12. Prediction Performance Comparison for Genome-Wide DNA Methylation Data Between BioM2 with Pathway-Stratified and Conventional Machine Learning(ML) Models**

Notes: The comparison involved 50 rounds of resampling tests, each randomly drawing 68 cases from the 359 available MDD cases. Each round was validated using 5 repetitions of 5-fold cross-validation. Bold markers (round 12, 19) indicate worse prediction performance by BioM2. In 48 out of 50 rounds, BioM2 outperformed the conventional model.

| **Permutation test** | **Models** | **AUC** | **PCCs** | **BAC** | **PRAUC** |
| --- | --- | --- | --- | --- | --- |
| round_1 | BioM2 | 0.715 | 0.387 | 0.648 | 0.695 |
|  | Conventional ML | 0.647 | 0.251 | 0.606 | 0.607 |
| round_2 | BioM2 | 0.712 | 0.359 | 0.664 | 0.687 |
|  | Conventional ML | 0.663 | 0.280 | 0.631 | 0.639 |
| round_3 | BioM2 | 0.731 | 0.403 | 0.668 | 0.725 |
|  | Conventional ML | 0.699 | 0.335 | 0.662 | 0.658 |
| round_4 | BioM2 | 0.689 | 0.308 | 0.655 | 0.694 |
|  | Conventional ML | 0.653 | 0.258 | 0.625 | 0.662 |
| round_5 | BioM2 | 0.660 | 0.264 | 0.620 | 0.635 |
|  | Conventional ML | 0.630 | 0.204 | 0.596 | 0.606 |
| round_6 | BioM2 | 0.679 | 0.307 | 0.622 | 0.640 |
|  | Conventional ML | 0.642 | 0.251 | 0.590 | 0.605 |
| round_7 | BioM2 | 0.668 | 0.313 | 0.604 | 0.683 |
|  | Conventional ML | 0.667 | 0.308 | 0.649 | 0.653 |
| round_8 | BioM2 | 0.717 | 0.385 | 0.667 | 0.697 |
|  | Conventional ML | 0.677 | 0.288 | 0.644 | 0.632 |
| round_9 | BioM2 | 0.701 | 0.364 | 0.672 | 0.708 |
|  | Conventional ML | 0.649 | 0.251 | 0.614 | 0.622 |
| round_10 | BioM2 | 0.615 | 0.184 | 0.586 | 0.592 |
|  | Conventional ML | 0.603 | 0.164 | 0.568 | 0.584 |
| round_11 | BioM2 | 0.789 | 0.496 | 0.721 | 0.795 |
|  | Conventional ML | 0.768 | 0.460 | 0.690 | 0.747 |
| **round_12** | BioM2 | 0.744 | 0.435 | 0.679 | 0.739 |
|  | Conventional ML | 0.751 | 0.418 | 0.698 | 0.790 |
| round_13 | BioM2 | 0.605 | 0.166 | 0.572 | 0.596 |
|  | Conventional ML | 0.571 | 0.115 | 0.550 | 0.561 |
| round_14 | BioM2 | 0.680 | 0.338 | 0.601 | 0.707 |
|  | Conventional ML | 0.654 | 0.274 | 0.617 | 0.657 |
| round_15 | BioM2 | 0.729 | 0.388 | 0.669 | 0.696 |
|  | Conventional ML | 0.693 | 0.325 | 0.663 | 0.644 |
| round_16 | BioM2 | 0.655 | 0.266 | 0.611 | 0.612 |
|  | Conventional ML | 0.606 | 0.186 | 0.565 | 0.575 |
| round_17 | BioM2 | 0.747 | 0.432 | 0.680 | 0.734 |
|  | Conventional ML | 0.720 | 0.344 | 0.604 | 0.713 |
| round_18 | BioM2 | 0.670 | 0.288 | 0.624 | 0.657 |
|  | Conventional ML | 0.664 | 0.285 | 0.618 | 0.644 |
| **round_19** | BioM2 | 0.667 | 0.296 | 0.645 | 0.630 |
|  | Conventional ML | 0.685 | 0.284 | 0.575 | 0.661 |
| round_20 | BioM2 | 0.639 | 0.247 | 0.583 | 0.648 |
|  | Conventional ML | 0.616 | 0.247 | 0.622 | 0.612 |
| round_21 | BioM2 | 0.661 | 0.266 | 0.620 | 0.654 |
|  | Conventional ML | 0.618 | 0.216 | 0.603 | 0.608 |
| round_22 | BioM2 | 0.674 | 0.304 | 0.626 | 0.663 |
|  | Conventional ML | 0.607 | 0.185 | 0.582 | 0.586 |
| round_23 | BioM2 | 0.683 | 0.310 | 0.635 | 0.664 |
|  | Conventional ML | 0.672 | 0.271 | 0.620 | 0.627 |
| round_24 | BioM2 | 0.767 | 0.462 | 0.686 | 0.766 |
|  | Conventional ML | 0.740 | 0.416 | 0.661 | 0.721 |
| round_25 | BioM2 | 0.627 | 0.217 | 0.584 | 0.591 |
|  | Conventional ML | 0.616 | 0.151 | 0.574 | 0.571 |
| round_26 | BioM2 | 0.657 | 0.256 | 0.602 | 0.627 |
|  | Conventional ML | 0.627 | 0.200 | 0.599 | 0.589 |
| round_27 | BioM2 | 0.736 | 0.416 | 0.674 | 0.746 |
|  | Conventional ML | 0.688 | 0.336 | 0.638 | 0.680 |
| round_28 | BioM2 | 0.667 | 0.295 | 0.629 | 0.667 |
|  | Conventional ML | 0.667 | 0.291 | 0.624 | 0.675 |
| round_29 | BioM2 | 0.712 | 0.363 | 0.648 | 0.714 |
|  | Conventional ML | 0.667 | 0.298 | 0.626 | 0.661 |
| round_30 | BioM2 | 0.769 | 0.473 | 0.688 | 0.764 |
|  | Conventional ML | 0.745 | 0.426 | 0.679 | 0.714 |
| round_31 | BioM2 | 0.660 | 0.274 | 0.621 | 0.667 |
|  | Conventional ML | 0.643 | 0.277 | 0.596 | 0.654 |
| round_32 | BioM2 | 0.696 | 0.335 | 0.658 | 0.673 |
|  | Conventional ML | 0.662 | 0.264 | 0.628 | 0.619 |
| round_33 | BioM2 | 0.703 | 0.339 | 0.645 | 0.689 |
|  | Conventional ML | 0.675 | 0.300 | 0.629 | 0.657 |
| round_34 | BioM2 | 0.700 | 0.367 | 0.646 | 0.682 |
|  | Conventional ML | 0.658 | 0.285 | 0.617 | 0.634 |
| round_35 | BioM2 | 0.769 | 0.461 | 0.715 | 0.790 |
|  | Conventional ML | 0.708 | 0.352 | 0.648 | 0.711 |
| round_36 | BioM2 | 0.639 | 0.230 | 0.599 | 0.628 |
|  | Conventional ML | 0.603 | 0.169 | 0.588 | 0.596 |
| round_37 | BioM2 | 0.647 | 0.265 | 0.606 | 0.661 |
|  | Conventional ML | 0.607 | 0.206 | 0.560 | 0.636 |
| round_38 | BioM2 | 0.779 | 0.484 | 0.702 | 0.781 |
|  | Conventional ML | 0.699 | 0.340 | 0.642 | 0.673 |
| round_39 | BioM2 | 0.792 | 0.513 | 0.721 | 0.794 |
|  | Conventional ML | 0.747 | 0.440 | 0.691 | 0.715 |
| round_40 | BioM2 | 0.716 | 0.377 | 0.649 | 0.701 |
|  | Conventional ML | 0.681 | 0.317 | 0.630 | 0.675 |
| round_41 | BioM2 | 0.677 | 0.326 | 0.599 | 0.700 |
|  | Conventional ML | 0.652 | 0.272 | 0.582 | 0.664 |
| round_42 | BioM2 | 0.759 | 0.450 | 0.693 | 0.742 |
|  | Conventional ML | 0.738 | 0.408 | 0.656 | 0.720 |
| round_43 | BioM2 | 0.627 | 0.210 | 0.599 | 0.603 |
|  | Conventional ML | 0.591 | 0.155 | 0.572 | 0.558 |
| round_44 | BioM2 | 0.694 | 0.339 | 0.638 | 0.684 |
|  | Conventional ML | 0.646 | 0.259 | 0.586 | 0.628 |
| round_45 | BioM2 | 0.780 | 0.484 | 0.707 | 0.761 |
|  | Conventional ML | 0.750 | 0.436 | 0.677 | 0.727 |
| round_46 | BioM2 | 0.805 | 0.531 | 0.726 | 0.811 |
|  | Conventional ML | 0.765 | 0.470 | 0.680 | 0.783 |
| round_47 | BioM2 | 0.650 | 0.260 | 0.619 | 0.663 |
|  | Conventional ML | 0.637 |  | 0.605 | 0.651 |
| round_48 | BioM2 | 0.728 | 0.378 | 0.668 | 0.670 |
|  | Conventional ML | 0.674 | 0.302 | 0.622 | 0.633 |
| round_49 | BioM2 | 0.787 | 0.503 | 0.724 | 0.787 |
|  | Conventional ML | 0.770 | 0.464 | 0.690 | 0.754 |
| round_50 | BioM2 | 0.665 | 0.275 | 0.632 | 0.630 |
|  | Conventional ML | 0.641 | 0.249 | 0.623 | 0.610 |

**Supplementary Table 13. Prediction Performance Comparison for Genome-Wide DNA Methylation Data Between BioM2 with Pathway-Stratified and Conventional Machine Learning Models (Without Sample Selection by MatchIt Package)**

Notes: Due to data imbalance (MDD=361, HC=68), we used the borderline-SMOTE algorithm to balance the classes (MDD=361, HC=356). This was implemented using the BLSMOTE (default) function of the smotefamily package (version 1.3.1)[1]. The BioM2 base model used is 'L2-regularized Logistic Regression' with the following settings: stage-1 cutoff at 0.3, top 1000 unmapped features, and stage-2 cutoff at 1.0. Performance was validated using 10 repetitions of 5-fold cross-validation.

| **Model** | **AUC, mean±SD** | **BAC, mean±SD** | **PRAUC, mean±SD** | **PCC, mean±SD** |
| --- | --- | --- | --- | --- |
| ***BioM2(GO_BP)*** | ***0.981±0.003*** | 0.947±0.006 | ***0.986±0.003*** | ***0.912±0.007*** |
| Decision Tree | 0.925±0.010 | 0.888±0.012 | 0.923±0.016 | 0.784±0.023 |
| Elastic Net | 0.970±0.005 | 0.934±0.004 | 0.975±0.008 | 0.885±0.005 |
| Gaussian Process | 0.964± 0.003 | 0.926±0.003 | 0.974±0.003 | 0.872±0.003 |
| Generalized Boosted Model | 0.965±0.005 | 0.926±0.004 | 0.974±0.003 | 0.876±0.005 |
| k-Nearest Neighbor | 0.975±0.006 | 0.925±0.006 | 0.972±0.008 | 0.900±0.010 |
| L2-regularized Logistic Regression | 0.978±0.006 | ***0.949±0.002*** | 0.984±0.003 | 0.909±0.005 |
| Logistic Regression | 0.675±0.011 | 0.741±0.011 | 0.551±0.009 | 0.517±0.021 |
| Naive Bayes Model | 0.921±0.001 | 0.918±0.001 | 0.949±0.001 | 0.848±0.002 |
| Random Forest | 0.980±0.003 | 0.918±0.001 | 0.984±0.002 | 0.872±0.002 |
| Support Vector Machine | 0.978±0.004 | 0.936±0.005 | 0.983±0.002 | 0.898±0.005 |

**Supplementary Table 14. Prediction Performance Comparison for Genome-Wide Gene Expression Data Between BioM2 with Pathway-Stratified and Conventional Machine Learning Models (Without Sample Selection by MatchIt Package)**

Notes: The comparison involves data from MDD patients (n=69) and healthy controls (n=91). The BioM2 base model used is 'Random Forest' with the following settings: stage-1 cutoff at 0.1, top 300 unmapped features, and stage-2 cutoff at 1.0. Performance was validated using 10 repetitions of 5-fold cross-validation.

| **Model** | **AUC, mean±SD** | **BAC, mean±SD** | **PRAUC, mean±SD** | **PCC, mean±SD** |
| --- | --- | --- | --- | --- |
| ***BioM2(GO_BP)*** | ***0.606±0.020*** | ***0.590± 0.019*** | ***0.542±0.033*** | ***0.187±0.037*** |
| Decision Tree | 0.525±0.042 | 0.522±0.035 | 0.467±0.036 | 0.042±0.072 |
| Elastic Net | 0.503±0.024 | 0.508±0.018 | 0.449±0.019 | NA |
| Naive Bayes Model | 0.602±0.024 | 0.580±0.019 | 0.525±0.027 | 0.162±0.036 |
| L2-regularized Logistic Regression | 0.530±0.023 | 0.536±0.025 | 0.479±0.023 | 0.056±0.035 |
| Random Forest | 0.589±0.028 | 0.576±0.027 | 0.534±0.024 | 0.142±0.042 |
| Gaussian Process | 0.605±0.026 | 0.577±0.022 | 0.535±0.020 | 0.161±0.036 |
| Generalized Boosted Model | 0.546±0.021 | 0.531±0.023 | 0.494±0.027 | 0.077±0.032 |
| k-Nearest Neighbor | 0.569±0.038 | 0.529±0.026 | 0.517±0.029 | 0.117±0.058 |
| Support Vector Machine | 0.598±0.027 | 0.582±0.017 | 0.536±0.023 | 0.167±0.036 |
| Logistic Regression | 0.503±0.046 | 0.495±0.038 | 0.440±0.033 | -0.005±0.081 |

**Supplementary Table 15. Top 20 most significant CpGs derived from top 10 pathways in genome-wide DNA methylation data.** *(P values are based on the Wilcoxon signed-rank test)*

| **ID** | **CpG_beg** | **CHR** | **RefGene_Name** | **RefGene_Group** | **CpG_Island** | **Pvalue** | **Correlation** |
| --- | --- | --- | --- | --- | --- | --- | --- |
| cg21843015 | 153284589 | 1 | PGLYRP3 | TSS1500 |  | 2.20×10^-8^ | -0.472 |
| cg18511798 | 2018148 | 11 | H19 | Body;TSS200 | Island | 5.51×10^-7^ | 0.419 |
| cg24577455 | 2069011 | 1 | PRKCZ | 5URT;Body | S_Shelf | 1.27×10^-6^ | 0.372 |
| cg01868499 | 89419218 | 10 | PAPSS2 | TSS1500 | N_Shore | 1.46×10^-6^ | 0.399 |
| cg15394860 | 2017083 | 11 | H19 | Body;3UTR;1stExon | S_Shelf | 2.25×10^-6^ | -0.403 |
| cg07275305 | 31227883 | 16 | TRIM72 | Body;3UTR | Island | 5.24×10^-6^ | 0.386 |
| cg00637015 | 31468125 | 1 | PUM1 | Body |  | 5.40×10^-6^ | 0.390 |
| cg12121660 | 46621708 | 17 | HOXB2 | Body;TSS200 | S_Shore | 6.92×10^-6^ | -0.337 |
| cg26886965 | 207672695 | 1 | CR1 | Body;Body | S_Shelf | 6.92×10^-6^ | 0.390 |
| cg10377144 | 89419176 | 10 | PAPSS2 | TSS1500 | N_Shore | 6.92×10^-6^ | 0.367 |
| cg18483526 | 24162797 | 16 | PRKCB | Body |  | 7.90×10^-6^ | 0.357 |
| cg16967204 | 65710348 | 7 | TPST1 | Body |  | 9.29×10^-6^ | 0.356 |
| cg04650641 | 41697409 | 22 | ZC3H7B | TSS200 | Island | 1.01×10^-5^ | -0.365 |
| cg22172494 | 2017361 | 11 | H19 | Body;3UTR;1stExon | S_Shelf | 1.37×10^-5^ | 0.384 |
| cg27574595 | 25583273 | 3 | RARB | Body |  | 1.37×10^-5^ | -0.345 |
| cg10608169 | 37964629 | 9 | SHB | Body |  | 1.44×10^-5^ | -0.405 |
| cg02774129 | 58712061 | 1 | DAB1 | 5URT | N_Shelf | 1.50×10^-5^ | 0.360 |
| cg17667220 | 58717078 | 1 | DAB1 | TSS1500 | S_Shore | 1.51×10^-5^ | -0.363 |
| cg05010623 | 158485761 | 2 | ACVR1C | TSS1500 | S_Shore | 2.05×10^-5^ | -0.345 |
| cg22064635 | 111340571 | 3 | CD96 | Body |  | 2.15×10^-5^ | 0.362 |

**Supplementary Table 16. The existing evidence for the top genes harboring top CpGs from Supplementary Table 15.**

| **ID** | **RefGene_Name** | **Definition** | **Reference** |
| --- | --- | --- | --- |
| cg24577455 | PRKCZ | [protein kinase C zeta](https://www.ncbi.nlm.nih.gov/gene/5590) | [2-4] |
| cg00637015 | PUM1 | [pumilio RNA binding family member 1](https://www.ncbi.nlm.nih.gov/gene/9698) | [5] |
| cg26886965 | CR1 | [complement C3b/C4b receptor 1 (Knops blood group)](https://www.ncbi.nlm.nih.gov/gene/1378) | [6] |
| cg18483526 | PRKCB | [protein kinase C beta](https://www.ncbi.nlm.nih.gov/gene/5579) | [7, 8] |
| cg16967204 | TPST1 | [tyrosylprotein sulfotransferase 1](https://www.ncbi.nlm.nih.gov/gene/8460) | [9] |
| cg04650641 | ZC3H7B | [zinc finger CCCH-type containing 7B](https://www.ncbi.nlm.nih.gov/gene/23264) | [10] |
| cg27574595 | RARB | retinoic acid receptor beta | [11] |

**Supplementary Table 17. Top 20 most significant gene probes derived from top 10 pathways in genome-wide gene expression data. (***P values are based on the Wilcoxon signed-rank test)*

| **ID** | **Symbol** | **CHR** | **Cytoband** | **Pvalue** | **Correlation** |
| --- | --- | --- | --- | --- | --- |
| ILMN_1693604 | GRM2 | 3 | 3p21.1e | 1.163×10^-4^ | -0.314 |
| ILMN_1696488 | FGF23 | 12 | 12p13.32a | 2.340×10^-4^ | -0.287 |
| ILMN_1813625 | TRIM25 | 17 | 17q22c | 3.000×10^-4^ | 0.283 |
| ILMN_1747227 | ADORA1 | 1 | 1q32.1d-q32.1e | 4.798×10^-4^ | -0.302 |
| ILMN_1765649 | IRF3 | 19 | 19q13.33b | 5.033×10^-4^ | -0.275 |
| ILMN_1781155 | LYN | 8 | 8q12.1a | 8.044×10^-4^ | 0.216 |
| ILMN_1669317 | GPR77 | 19 | 19q13.32b-q13.32c | 1.193×10^-3^ | 0.278 |
| ILMN_1733155 | GIT1 | 17 | 17q11.2b | 1.229×10^-3^ | -0.278 |
| ILMN_1767365 | PAK1 | 11 | 11q14.1a | 1.674×10^-3^ | 0.230 |
| ILMN_1654319 | HAPLN3 | 15 | 15q26.1a | 2.264×10^-3^ | -0.231 |
| ILMN_1666933 | ASH2L |  | 8p12a | 2.571×10^-3^ | 0.239 |
| ILMN_1682459 | TUBB4 | 19 | 19p13.3a | 2.875×10^-3^ | -0.238 |
| ILMN_1765876 | ARHGAP24 | 4 | 4q21.23b-q21.3a | 3.256×10^-3^ | 0.210 |
| ILMN_2284998 | SP100 | 2 | 2q37.1a | 3.633×10^-3^ | 0.275 |
| ILMN_1669663 | BCR | 22 | 22q11.23a | 3.784×10^-3^ | -0.255 |
| ILMN_1676955 | TYK2 | 19 | 19p13.2b | 4.629×10^-3^ | 0.223 |
| ILMN_1726245 | TGFBR2 | 3 | 3p24.1a | 4.947×10^-3^ | 0.221 |
| ILMN_1803686 | ADA | 20 | 20q13.12a | 5.215×10^-3^ | -0.185 |
| ILMN_1664094 | P2RY13 | 3 | 3q25.1c | 5.569×10^-3^ | 0.200 |
| ILMN_1726597 | C6orf32 | 6 | 6p22.2b | 7.210×10^-3^ | 0.214 |

**Supplementary Table 18. The existing evidence for the top genes harboring top gene probes from Supplementary Table 17.**

| **ID** | **Symbol** | **Definition** | **Reference** |
| --- | --- | --- | --- |
| ILMN_1693604 | GRM2 | glutamate metabotropic receptor 2 | [12, 13] |
| ILMN_1696488 | FGF23 | [fibroblast growth factor 23](https://www.ncbi.nlm.nih.gov/gene/8074) | [14] |
| ILMN_1733155 | GIT1 | [GIT ArfGAP 1](https://www.ncbi.nlm.nih.gov/gene/28964) | [15] |
| ILMN_1767365 | PAK1 | [p21 (RAC1) activated kinase 1](https://www.ncbi.nlm.nih.gov/gene/5058) | [16] |
| ILMN_1682459 | TUBB4 | [tubulin beta 4B class IVb](https://www.ncbi.nlm.nih.gov/gene/10383) | [17] |
| ILMN_1676955 | TYK2 | [tyrosine kinase 2](https://www.ncbi.nlm.nih.gov/gene/7297) | [18] |
| ILMN_1726245 | TGFBR2 | [transforming growth factor beta receptor 2](https://www.ncbi.nlm.nih.gov/gene/7048) | [19, 20] |

**Reference**

1. Siriseriwan, W., *Smotefamily: A collection of oversampling techniques for class imbalance problem based on SMOTE.* R package version, 2019. **1**(1).

2. Shi, Y., et al., *Genetic variation in the calcium/calmodulin-dependent protein kinase (CaMK) pathway is associated with antidepressant response in females.* Journal of affective disorders, 2012. **136**(3): p. 558-566.

3. Verma, P. and M. Shakya, *Transcriptomics and sequencing analysis of gene expression profiling for major depressive disorder.* Indian Journal of Psychiatry, 2021. **63**(6): p. 549-553.

4. Lohoff, F.W., et al., *Epigenome-wide association study and multi-tissue replication of individuals with alcohol use disorder: evidence for abnormal glucocorticoid signaling pathway gene regulation.* Molecular psychiatry, 2021. **26**(6): p. 2224-2237.

5. Dong, H., et al., *Pumilio2 regulates synaptic plasticity via translational repression of synaptic receptors in mice.* Oncotarget, 2018. **9**(63): p. 32134.

6. Gibson, J., et al., *Assessing the presence of shared genetic architecture between Alzheimer’s disease and major depressive disorder using genome-wide association data.* Translational psychiatry, 2017. **7**(4): p. e1094-e1094.

7. Costas, J., et al., *Association study of 44 candidate genes with depressive and anxiety symptoms in post-partum women.* Journal of psychiatric research, 2010. **44**(11): p. 717-724.

8. Salvetat, N., et al., *A game changer for bipolar disorder diagnosis using RNA editing-based biomarkers.* Translational Psychiatry, 2022. **12**(1): p. 182.

9. Zhao, S., et al., *Identification of diagnostic markers for major depressive disorder using machine learning methods.* Frontiers in neuroscience, 2021. **15**: p. 645998.

10. Wu, W., et al., *Differential and spatial expression meta-analysis of genes identified in genome-wide association studies of depression.* Translational psychiatry, 2021. **11**(1): p. 8.

11. Mulvey, B. and J.D. Dougherty, *Transcriptional-regulatory convergence across functional MDD risk variants identified by massively parallel reporter assays.* Translational psychiatry, 2021. **11**(1): p. 403.

12. Dogra, S. and P.J. Conn, *Targeting metabotropic glutamate receptors for the treatment of depression and other stress-related disorders.* Neuropharmacology, 2021. **196**: p. 108687.

13. Mariani, N., et al., *Gene expression studies in Depression development and treatment: an overview of the underlying molecular mechanisms and biological processes to identify biomarkers.* Translational psychiatry, 2021. **11**(1): p. 354.

14. Xu, Y.H., et al., *Abnormalities in FGF family members and their roles in modulating depression‐related molecules.* European Journal of Neuroscience, 2021. **53**(1): p. 140-150.

15. Fuchsova, B., et al., *Altered expression of neuroplasticity-related genes in the brain of depressed suicides.* Neuroscience, 2015. **299**: p. 1-17.

16. Fuchsova, B., et al., *Expression of p21-activated kinases 1 and 3 is altered in the brain of subjects with depression.* Neuroscience, 2016. **333**: p. 331-344.

17. Kang, H.J., et al., *Decreased expression of synapse-related genes and loss of synapses in major depressive disorder.* Nature medicine, 2012. **18**(9): p. 1413-1417.

18. Zhang, H.-G., et al., *Depression compromises antiviral innate immunity via the AVP-AHI1-Tyk2 axis.* Cell Research, 2022. **32**(10): p. 897-913.

19. Zhang, K., et al., *Essential role of microglial transforming growth factor-β1 in antidepressant actions of (R)-ketamine and the novel antidepressant TGF-β1.* Translational psychiatry, 2020. **10**(1): p. 32.

20. Ma, L., et al., *A key role of miR-132-5p in the prefrontal cortex for persistent prophylactic actions of (R)-ketamine in mice.* Translational Psychiatry, 2022. **12**(1): p. 417.
